# In vitro and ex vivo metabolism of chemically diverse fructans by bovine rumen *Bifidobacterium* and *Lactobacillus* species

**DOI:** 10.1186/s42523-024-00328-1

**Published:** 2024-09-09

**Authors:** Marissa L. King, Xiaohui Xing, Greta Reintjes, Leeann Klassen, Kristin E. Low, Trevor W. Alexander, Matthew Waldner, Trushar R. Patel, D. Wade Abbott

**Affiliations:** 1 Lethbridge Research and Development Centre, Agriculture and Agri-Food Canada, Lethbridge, AB Canada; 2 Department of Chemistry and Biochemistry, University of Lethbridge, Lethbridge, AB Canada; 3 Microbial-Carbohydrate Interactions Group, Department of Biology/Chemistry, University of Bremen, Bremen, Germany; 4 Max Planck Institute for Marine Microbiology, Bremen, Germany; 5 Faculty of Veterinary Medicine, University of Calgary, Calgary, AB Canada

**Keywords:** Fructan, Linkage analysis, *Bifidobacterium*, *Lactobacillus*, Prebiotics, Rumen, Microbiome, Carbohydrate-active enzyme

## Abstract

**Background:**

Inulin and inulin-derived fructooligosaccharides (FOS) are well-known prebiotics for use in companion animals and livestock. The mechanisms by which FOS contribute to health has not been fully established. Further, the fine chemistry of fructan structures from diverse sources, such as graminan-type fructans found in cereal crops, has not been fully elucidated. New methods to study fructan structure and microbial responses to these complex carbohydrates will be key for evaluating the prebiotic potency of cereal fructans found in cattle feeds. As the rumen microbiome composition is closely associated with their metabolic traits, such as feed utilization and waste production, prebiotics and probiotics represent promising additives to shift the microbial community toward a more productive state.

**Results:**

Within this study, inulin, levan, and graminan-type fructans from winter wheat, spring wheat, and barley were used to assess the capacity of rumen-derived *Bifidobacterium boum*, *Bifidobacterium merycicum*, and *Lactobacillus vitulinus* to metabolize diverse fructans. Graminan-type fructans were purified and structurally characterized from the stems and kernels of each plant. All three bacterial species grew on FOS, inulin, and cereal crop fructans in pure cultures. *L. vitulinus* was the only species that could metabolize levan, albeit its growth was delayed. Fluorescently labelled polysaccharides (FLAPS) were used to demonstrate interactions with Gram-positive bacteria and confirm fructan metabolism at the single-cell level; these results were in agreement with the individual growth profiles of each species. The prebiotic potential of inulin was further investigated within naïve rumen microbial communities, where increased relative abundance of *Bifidobacterium* and *Lactobacillus* species occurred in a dose-dependent and temporal-related manner. This was supported by in situ analysis of rumen microbiota from cattle fed inulin. FLAPS probe derived from inulin and fluorescent in situ hybridization using taxon-specific probes confirmed that inulin interacts with Bifidobacteria and Lactobacilli at the single-cell level.

**Conclusion:**

This research revealed that rumen-derived Bifidobacteria and Lactobacilli vary in their metabolism of structurally diverse fructans, and that inulin has limited prebiotic potential in the rumen. This knowledge establishes new methods for evaluating the prebiotic potential of fructans from diverse plant sources as prebiotic candidates for use in ruminants and other animals.

**Supplementary Information:**

The online version contains supplementary material available at 10.1186/s42523-024-00328-1.

## Introduction

Fructans are fructose-rich oligosaccharides and polysaccharides produced for energy storage by some microbial species [1, 2] and approximately 15% of angiosperms [3]. Fructans have a terminal core of sucrose with an extended chain of fructosyl residues. Fructans can be described by their degree of polymerization (DP); fructooligosaccharides (FOS) have a DP < 10 and fructans contain 10 fructosyl residues or more. The classification of fructans is also based upon the positional chemistry of the glycosidic linkages and the glucosyl residue [4]. Linear fructans with a terminal glucosyl residue include inulin-type and levan-type fructans, which predominantly contain β-2,1 and β-2,6 glycosidic linkages, respectively. Neo-inulin and neo-levan-type fructans consist primarily of the same glycosidic linkages as their aforementioned counterparts, but possess an internal glycosyl residue. Graminan-type fructans are branched and have both β-2,1 and β-2,6 fructosyl linkages [5]. Currently, the prebiotic potential of graminan-type fructans is being investigated for applications in human health using immature wheat grain food products [6]; however, the effect of these graminan-type fructans has yet to be studied in a purified form.

Many fructan-storing plants are of great economic importance, including cereals (e.g., wheat, barley, rye) [7], fruits and vegetables (chicory, onion, Jerusalem artichoke, agave) [8], and forage grasses (ryegrass, fescue) [9]. The ability of plants to store fructans is closely associated with stress tolerance; fructans have been shown to act as osmoregulators during harsh growing conditions, such as drought, extreme cold, and high salinity [10]. Within cereal crops, fructans are abundant during the immature growth stage before starch accumulation occurs [11]. Fructan metabolism within wheat has been well documented [12]. Accumulation in wheat kernels predominantly occurs during the first 14 days after anthesis (DAA) [7] with an average DP of 7.3 ± 0.4 [12]. These are primarily graminan-type fructans, although some fructan neoseries have also been reported [11].

Inulin and inulin-derived FOS are well-known prebiotics for use in humans [13] and monogastric animals, such as poultry, swine, and calves [12, 14]. Beneficial health effects correlated with inulin or FOS supplementation include increased proliferation and activity of Bifidobacteria and Lactobacilli, reduced pathogen prevalence through competitive inhibition, and improved host immunity. Despite these beneficial effects, the abundance of Bifidobacteria and Lactobacilli remains relatively low in cattle and other ruminants after the weaning period [13, 14]. A fully developed rumen houses a complex and dynamic microbial ecosystem, with the core microbiome consisting of Bacteroidota (formerly Bacteroidetes), Bacillota (formerly Firmicutes), Pseudomondata (formerly Proteobacteria), and Fibrobacter [15]. The digestive capabilities of ruminants rely on the rumen microbiome, which possesses a remarkable diversity of carbohydrate-active enzymes (CAZymes) encoded within the genomes of rumen bacteria, fungi, and protists [16]. CAZymes belonging to the glycoside hydrolase family 32 (GH32) are known to cleave the β-2,1 and/or β-2,6 linkages present within different fructan polysaccharides [17]. GH32s can display differing modes of activity, such as β-fructofuranosidases, endo- and exo-acting inulinases and levanases, or non-specific fructan β-fructosidases; therefore, high-resolution bioinformatics or biochemical approaches are required to accurately predict or characterize their function, respectively. Additionally, CAZymes can also contain a carbohydrate-binding module (CBM), which potentiate CAZyme activity by targeting the enzyme to the substrate or concentrating it on the substrate’s surface [18, 19].

Here, we purify fructans from the stem and kernel fractions of immature cereal crops (7 DAA) and structurally characterize them using glycosidic linkage analysis. Further, we investigate the ability of three rumen-derived bacterial species, *Bifidobacterium boum*, *Bifidobacterium merycicum*, and *Lactobacillus vitulinus*, to catabolize structurally diverse fructan carbohydrates using bioinformatics, nutrient-restrictive liquid cultures, and FLAPS, a next-generation physiology approach to directly visualize cell-polysaccharide interactions [20–23]. The prebiotic potential of inulin was investigated ex vivo using rumen microbial communities supplemented with inulin, whereby 16 S rRNA metagenomic sequencing and FLAPS coupled to fluorescence in situ hybridization (FISH) were used to identify bacteria that directly interact with inulin. Finally, the rumen microbiota associated with in situ digestion of an inulin-supplement diet was evaluated in beef cattle. Investigating the relationship between potential prebiotics and beneficial bacteria such as Bifidobacteria and Lactobacilli, within an environment where there are found in low amounts, may increase their relative abundance and beneficial effects towards the host.

## Results

**Fig. 1:**
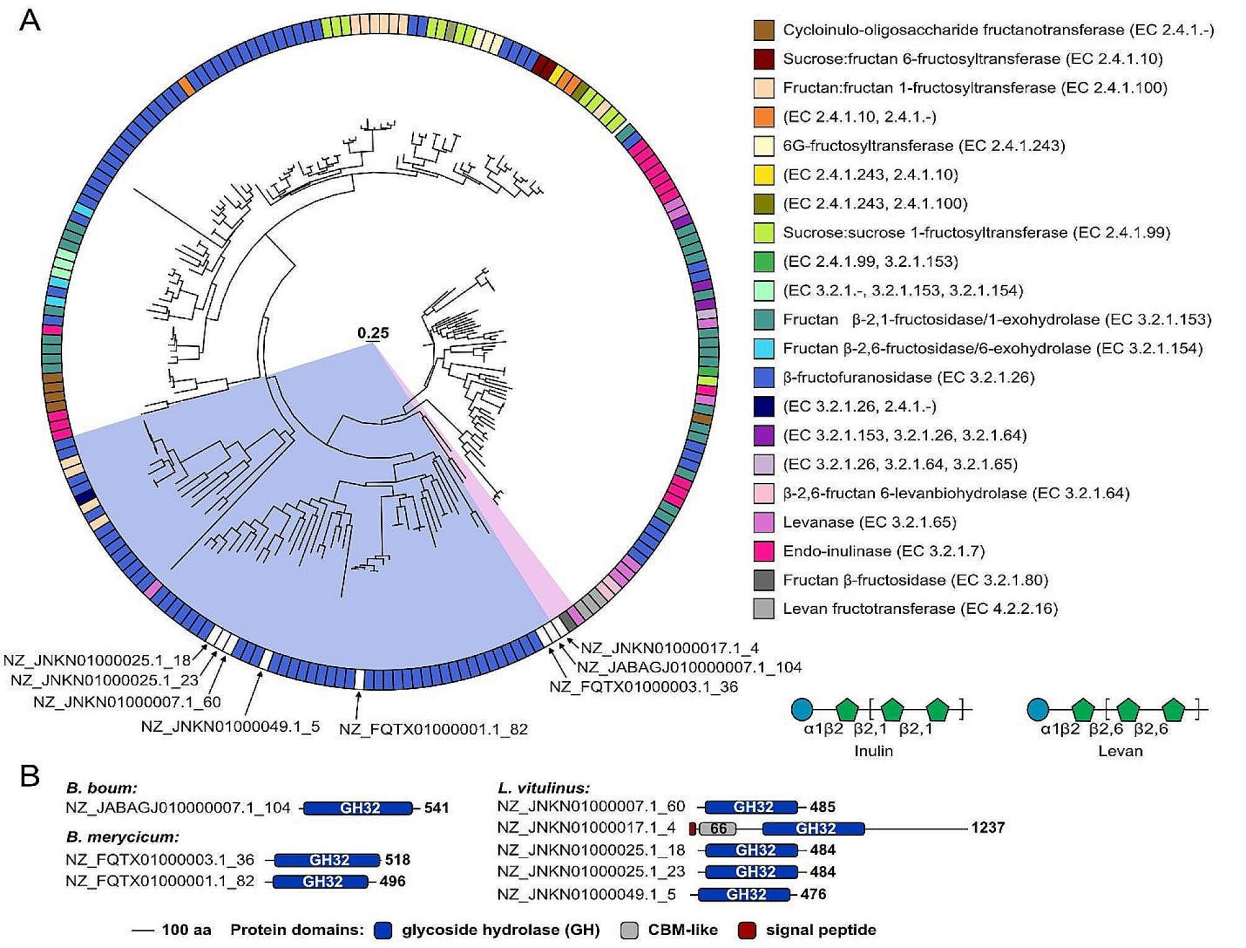
Predicted enzymatic activities of GH32s belonging to rumen-derived *Bifidobacterium* and *Lactobacillus* species. **(A)** Phylogenetic tree of characterized GH32s and sequences encoding GH32s from rumen-derived *Bifidobacterium* and *Lactobacillus* species, generated with SACCHARIS [26]. **(B)** GH32 protein modules for each species, analyzed using InterProScan [28]

To determine if rumen-derived *Bifidobacterium* and *Lactobacillus* species-derived probiotics had the potential to metabolize diverse fructans, the genomes of *B. boum* (ATCC 27,917), *B. merycicum* (ATCC 49,391), and *L. vitulinus* (ATCC 27,783) were retrieved from the NCBI assembly database and analyzed using dbCAN2 [24]. The genome of *L. vitulinus* encodes five GH32s, whereas *B. merycicum* has two GH32s, and *B. boum* a single GH32. MUSCLE [25] alignment of these GH32 sequences determined that the highest level of sequence conservation was amongst three GH32s encoded within *L. vitulinus* (NZ_JNKN01000007.1_60, NZ_JNKN01000025.1_18, and NZ_JNKN01000025.1_23; 50.5–63.5% similarity) (Table S1). There was 51.3% identity (486 aa) between the GH32 of *B. boum* (NZ_JABAGJ010000007.1_104) and a GH32 of *B. merycicum* (NZ_FQTX01000003.1_36). These predicted GH32s were inserted into a phylogenetic tree constructed with previously characterized GH32s using SACCHARIS [26]. Most of the GH32s from the three species were predicted to be β-fructofuranosidases (EC 3.2.1.26, Fig. 1A). However, one GH32 belonging to *L. vitulinus* (NZ_JNKN01000017.1_4, 20.0-22.4% similarity, Table S1) partitioned with an endo-levanase (EC 3.2.1.65) and a fructan β-fructosidase (EC 3.2.1.80) from *Streptococcus mutans* GS-5 (fruA, GenBank AAA36889.1) (Fig. 1A). Analysis using InterProScan [27] showed that the *L. vitulinus* NZ_JNKN01000017.1_4 GH32 also contained a N-terminal CBM66 and was predicted to be secreted (Fig. 1B).

### Catabolism of commercial fructans by rumen-derived bacteria with probiotic properties

**Fig. 2:**
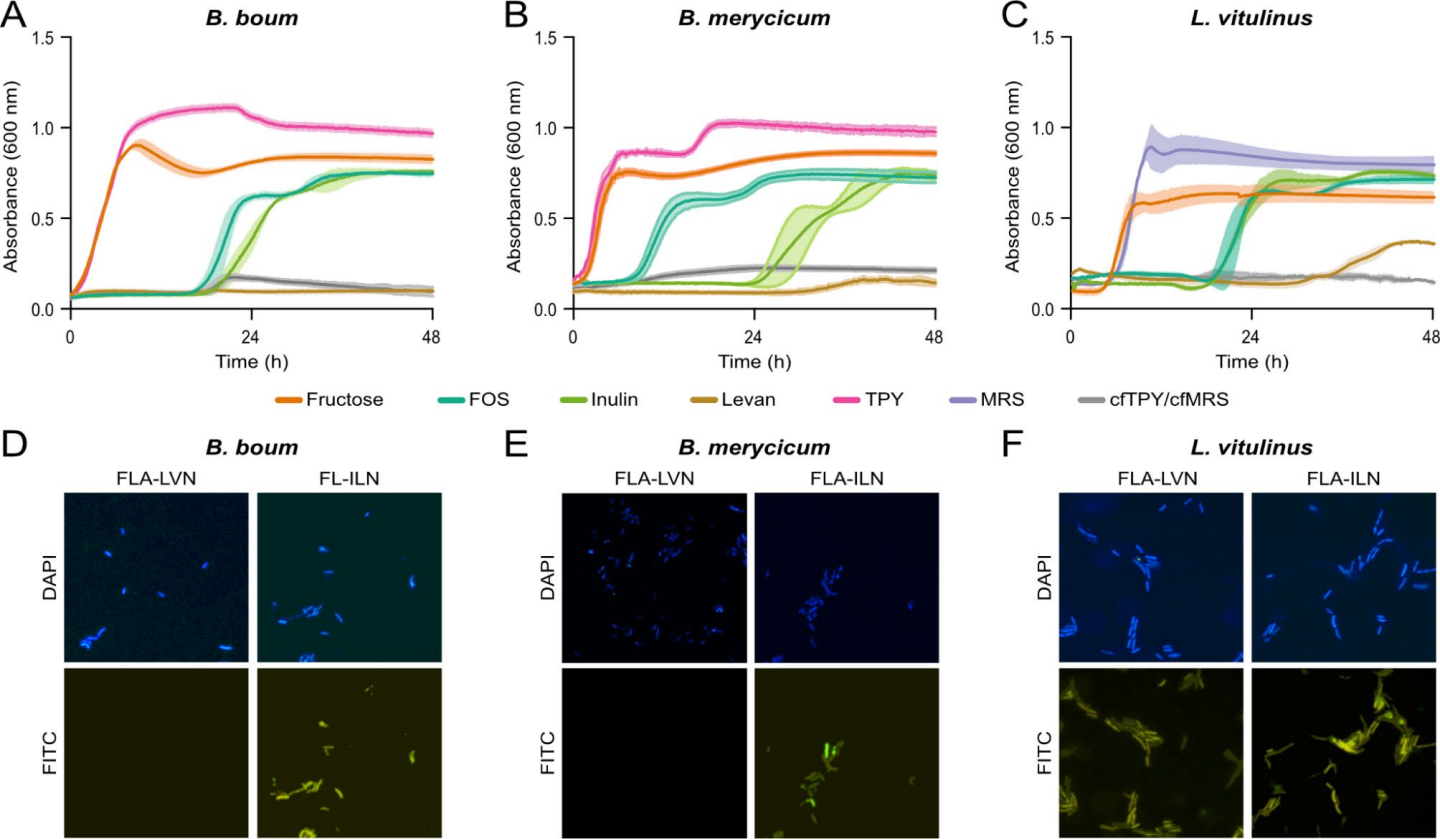
Utilization of commercialized fructans by rumen-derived *Bifidobacterium* and *Lactobacillus* species. Growth profiles of **(A)** *B. boum*, **(B)** *B. merycicum*, and **(C)** *L. vitulinus* grown on 0.5% fructose, fructooligosaccharides (FOS), inulin, or levan-cfTPY/ cfMRS, or in rich media (TPY or MRS). Epifluorescence visualization of 0.2% fluorescently labelled inulin (FLA-ILN) and 0.2% fluorescently labelled levan (FLA-LVN) interactions in pure cultures of **(D)** *B. boum*, **(E)** *B. merycicum*, and **(F)** *L. vitulinus* after a 1 d incubation. Cells were primed with 0.5% unlabelled inulin or levan for 16 h prior to FLAPS. Cells co-stained with DAPI, and visualized with the DAPI and fluorescein isothiocyanate (FITC) channels

Rumen bacteria encoding GH32s predicted to hydrolyze fructans were cultured to evaluate their ability to catabolize commercial fructans, which included inulin and FOS derived from chicory root, and levan synthesized by *Erwinia herbicola.* All three species could grow on FOS and inulin when used as the sole carbohydrate source (Fig. 2A-C). *B. merycicum* displayed biphasic growth on and differential responses to the two β-2,1 containing fructans, with a shorter lag phase for FOS than inulin (Fig. 2B, 12 h). Whereas *B. boum* and *L. vitulinus* had similar growth profiles when cultured on FOS and inulin. Of the three species, only *L. vitulinus* was able to utilize levan (Fig. 2C); although growth followed an extended lag phase of approximately 36 h.

Interactions between commercial fructan polysaccharides and *Bifidobacterium* and *Lactobacillus* species were further assessed using fluorescently labelled (FLA) inulin (FLA-ILN) and levan (FLA-LVN) for the direct visualization of metabolically active cells [20, 29]. In agreement with their respective growth curves, all three species showed positive interactions with FLA-ILN (Fig. 2D-E), while *L. vitulinus* was the only species to interact with FLA-LVN (Fig. 2E).

### Presence of Graminan-type fructans in immature cereal crops

To further assess the fructan-catabolizing abilities of the three bacterial species, fructans were purified from Canadian prairie spring wheat (cv. AC Andrew), hard red winter wheat (cv. AAC Coldfront), and barley (cv. AC Metcalfe) at an immature growth stage (7 DAA). Fructan quantification was conducted with a fructan assay kit (Megazyme Ltd., Ireland) for the kernel and stem portions of the three cereal crops [30], and assessed using HPAEC-PAD (Figure S1) and LC-MS (Figure S2). Comparison between the tissue types revealed that fructans are more abundant (> 4-fold) within the stem of spring wheat and barley when compared to kernels; whereas, similar amounts were observed in the two winter wheat samples tissues (Table 1). Fructan content was highest within barley stem (21.9 ± 0.71 g/100 g dry weight).

**Table 1:**
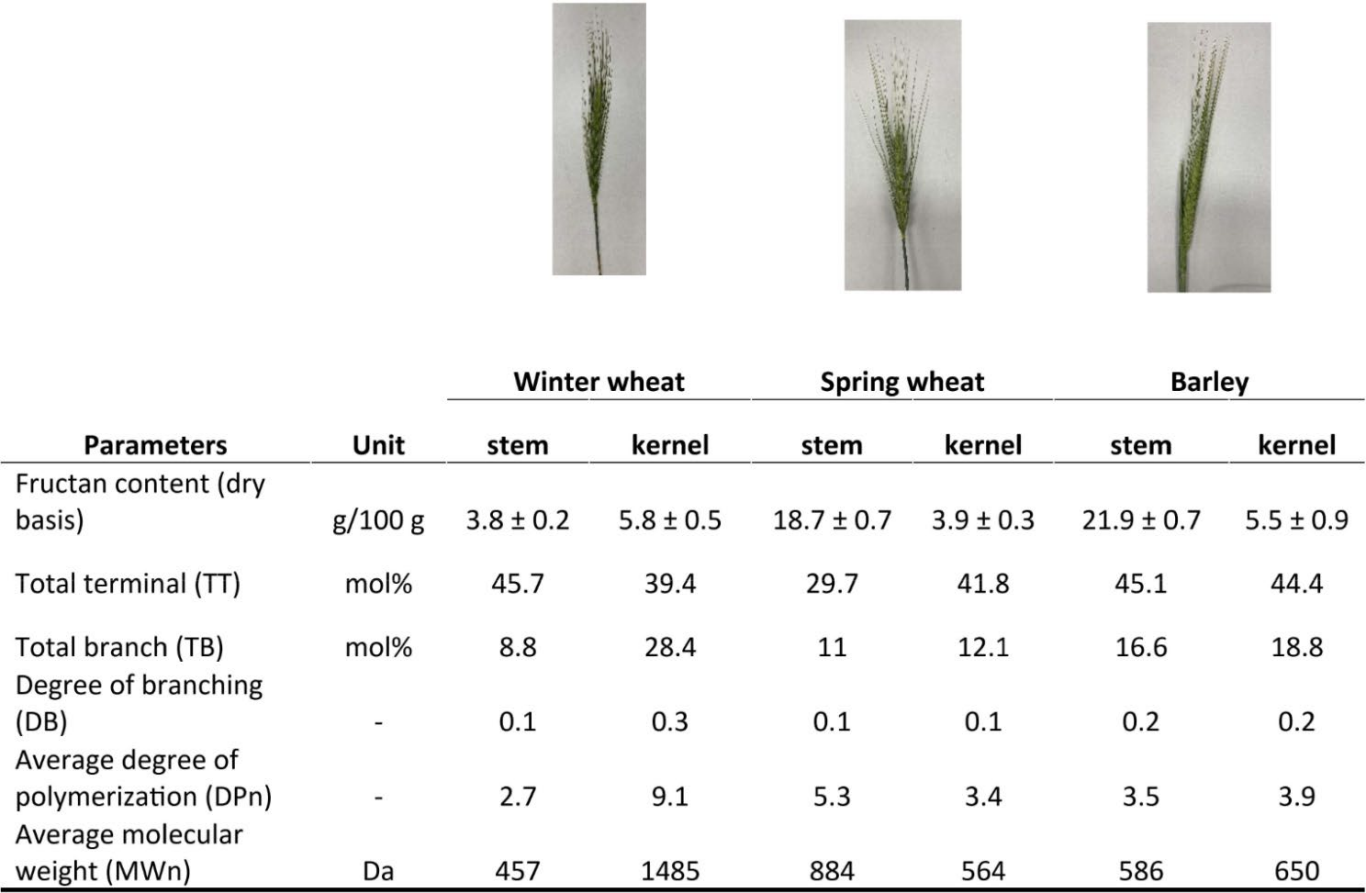
Abundance and structural characteristics of fructans purified from immature cereal crops. Fructans were purified from the kernel and stem sections of immature winter wheat, spring wheat, and barley (7 DAA)

Note DB is defined as average numbers of branches per monosaccharide unit. Calculations: DB = TT/100; DPn = 100/(TT-TB); MWn = 180×DPn-18×(DPn-1).

**Fig. 3:**
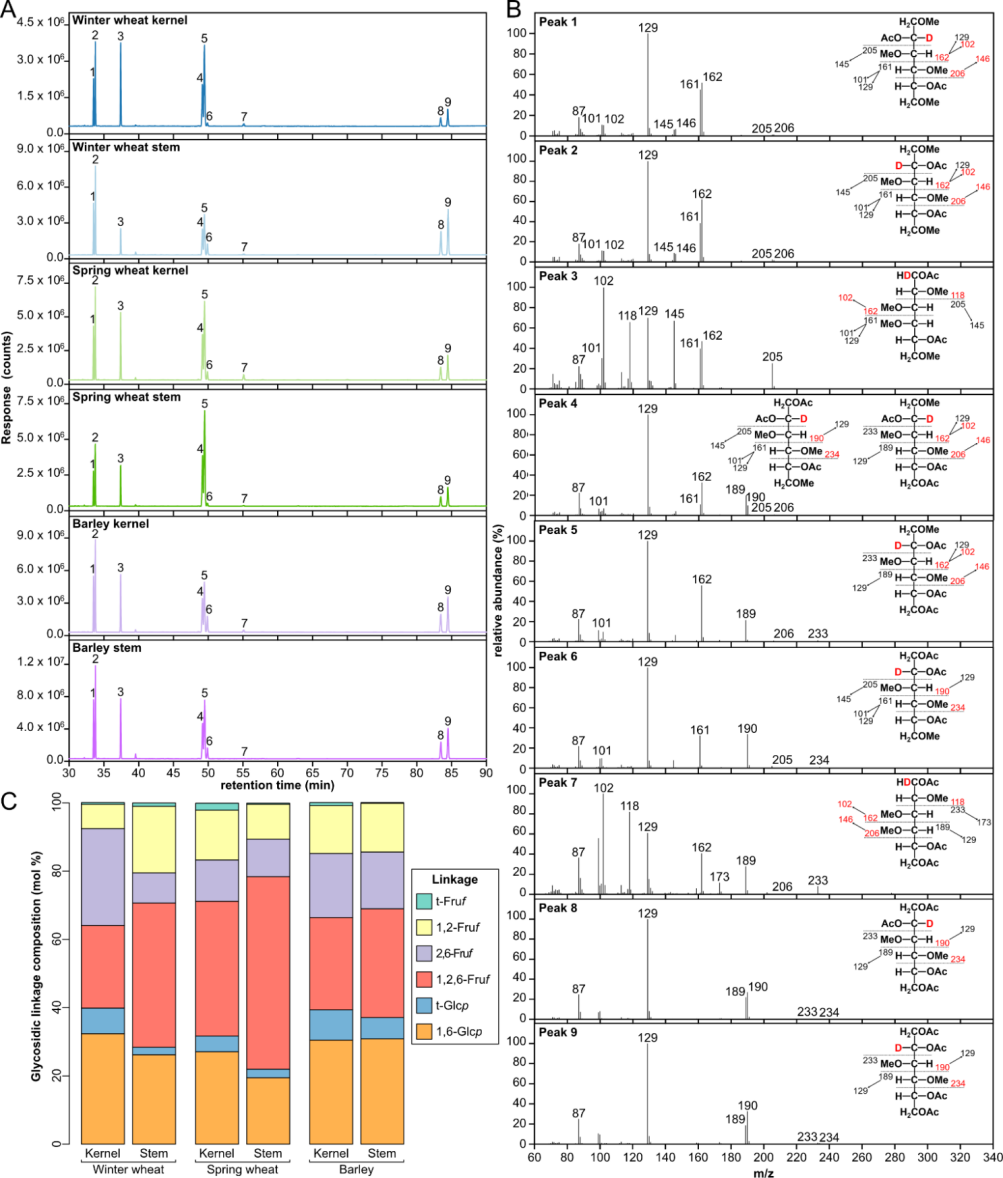
Glycosidic linkage analysis of fructans purified from immature cereal crops. **(A)** Total ion current (TIC) chromatograms of fructans purified from the kernel and stem sections of immature winter wheat (blue), spring wheat (green), and barley (purple) (7 DAA). **(B)** EI-MS spectra for peaks 1–9 with corresponding PMAA structures. **(C)** Composition of glycosidic linkages present in the six fructan samples

Methylation-GC-MS analysis was used to determine the relative composition of glycosidic linkages in fructans purified from the kernel and stems of winter wheat, spring wheat, and barley. Fructans were per-*O*-methylated, followed by weak acid hydrolysis, sodium borodeuteride reduction, and per-*O*-acetylation to generate deuterium-labeled PMAA derivatives. The PMAAs were well separated by the GC column as shown in the total ion current (TIC) chromatograms (Fig. 3A), identified by their EI-MS fragmentation patterns (Fig. 3B), and quantified as a relative composition based on TIC peak areas (Fig. 3C). Fructose, being a ketose, has C-2 as the anomeric carbon. Therefore, after sodium borohydride reduction, each fructose linkage generates a pair of C-2 deuterated glucitol and mannitol, which are distinguishable by EI-MS from their C-1 deuterated alditol counterparts derived from aldose sugar linkage (e.g., glucitol from glucose, mannitol from mannose). Detailed peak assignments of linkages from fructose and glucose are shown in Table S2. Results showed that t-Fru*f* and 2,6-Fru*f* were generally the two most abundant linkages in immature cereal crop fructans, with the exception of winter wheat kernels, as this fraction contained a high abundance of 1,2,6-Fru*f* as well (Fig. 3C).

### Catabolism of immature cereal crop fructans by rumen-derived bacteria with probiotic properties

**Fig. 4:**
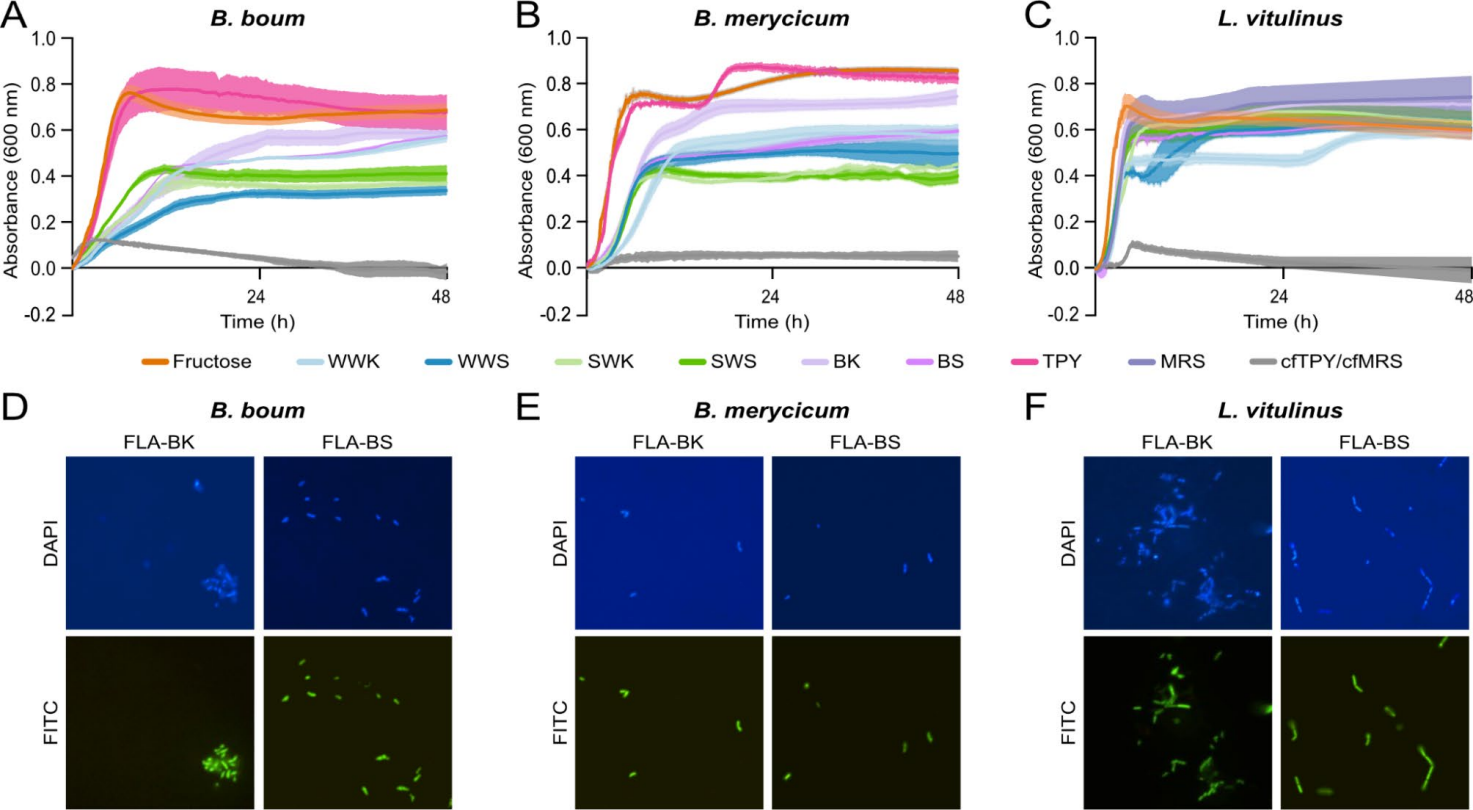
Utilization of fructans purified from immature cereal crops by rumen-derived *Bifidobacterium* and *Lactobacillus* species. Growth profiles of **(A)** *B. boum*, **(B)** *B. merycicum*, and **(C)** *L. vitulinus* grown on 0.5% fructans isolated from the kernel and stem sections of winter wheat, spring wheat, and barley samples (7 DAA) in cfTPY/ cfMRS, alongside growth in rich media (TPY or MRS). Epifluorescence visualization of 0.2% fluorescently labelled barley kernel fructans (FLA-BK) and 0.2% fluorescently labelled barley stem fructans (FLA-BS) interactions in pure cultures of **(D)** *B. boum*, **(E)** *B. merycicum*, and **(F)** *L. vitulinus* after a 1 h incubation. Cells co-stained with DAPI, and visualized with the DAPI and fluorescein isothiocyanate (FITC) channels

Each bacterial species was cultured to assess their fructan-catabolizing ability using fructans purified in-house from the kernel and stem fractions of immature winter wheat, spring wheat, and barley (7 DAA). All three species showed utilization of fructans from each cereal crop fraction within the first 12 h of incubation (Fig. 4A-C). Growth of *B. boum* (Fig. 4A) and *B. merycicum* (Fig. 4B) on the six fructan samples revealed differences in maximal OD_600nm_ by crop type. For *L. vitulinus*, growth on any of the purified fructans resulted in similar growth profiles to fructose as the sole carbon source, and growth on winter wheat fructans resulted in biphasic growth. Interactions between purified crop fructans and *Bifidobacterium* and *Lactobacillus* species were assessed using fluorescently labelled (FLA) fructans from immature barley kernel (FLA-BK) and barley stem (FLA-BS) for direct visualization. In agreement with their respective growth curves, all three species showed positive interactions with FLA-BK and FLA-BS (Fig. 2D-F).

### Inulin utilization in artificial rumen systems seeded with naïve rumen microbial communities

**Fig. 5:**
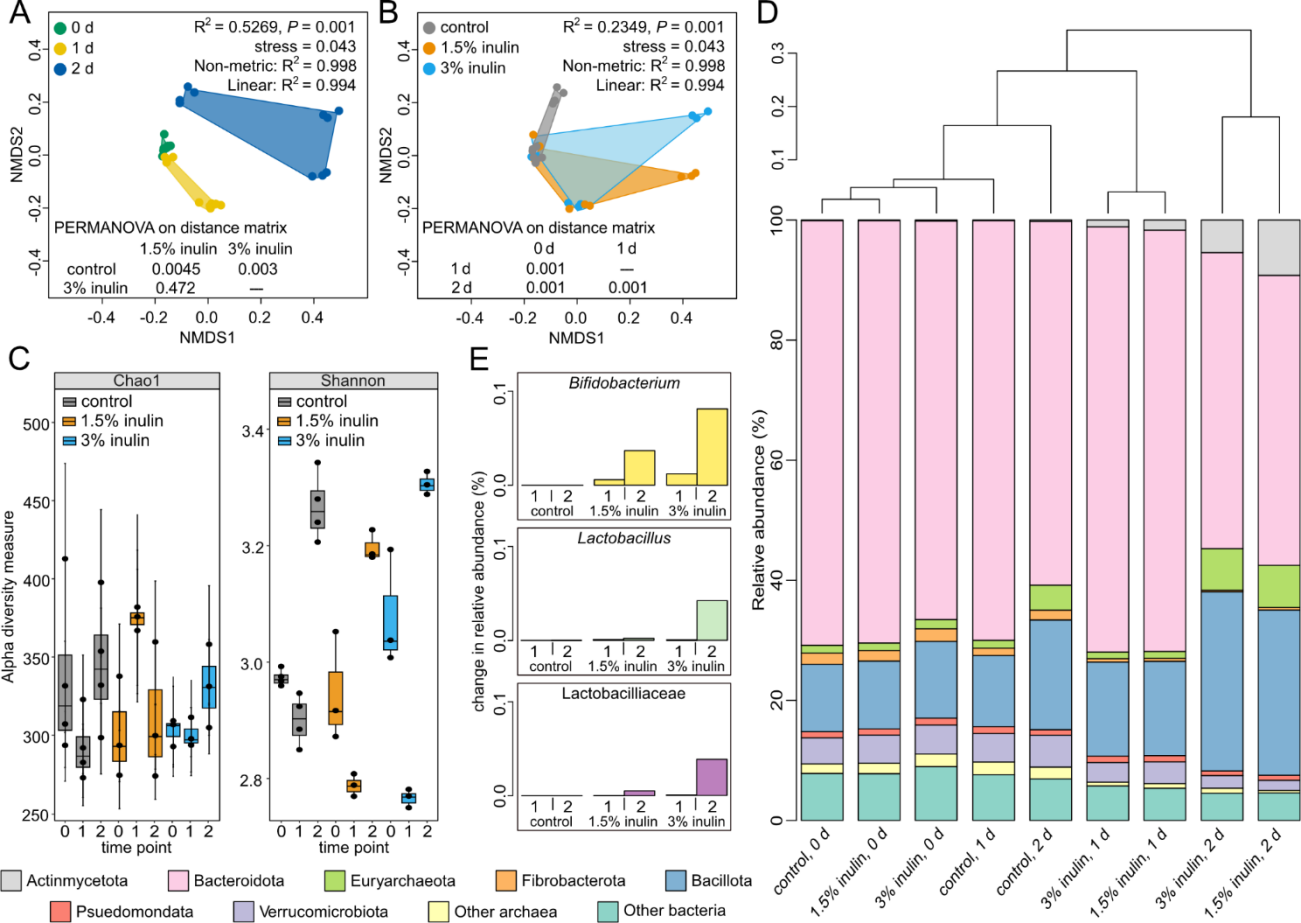
Ex vivo community analysis of rumen microbial communities enriched with inulin. **(A)** Non-metric multi-dimensional scaling (NMDS) plot comparing the community composition at different time points: 0 h (green), 1 d (yellow), and 2 d (blue). **(B)** NMDS plot comparing the effect inulin enrichment has on the community composition for control (grey), 1.5% inulin (light blue), and 3% inulin (orange). **(C)** Alpha-diversity (Chao1 and Shannon indices) in rumen microbial communities enriched with inulin over time. **(D)** Dendrogram showing similarity clustering of rumen microbial community compositions between different treatments and time points. Bar plots underneath display the relative abundance of common ruminal bacterial and archaeal phyla in rumen samples. **(E)** Percent change in abundance of *Bifidobacterium* species (yellow), *Lactobacillus* species (mint), and other Lactobacillaceae members (purple)

The rumen microbiome is highly efficient in converting host-indigestible plant fibre into accessible energy sources [31]. In this light, it is unclear whether prebiotics such as inulin added at low inclusion rates have an effect within this ecosystem [32]. To investigate the impact of inulin supplementation on the rumen microbial community ex vivo, rumen samples from a cow fed a background diet (i.e., not supplemented with inulin) were enriched with inulin at 1.5% or 3%, and then evaluated for 2 days. The composition of rumen microbial communities significantly changed between time points (R^2^ = 0.5269, *P* = 0.001; Fig. 5A) and treatment conditions (R^2^ = 0.2349, *P* = 0.001; Fig. 5B) based on the Bray-Curtis dissimilarity matrices. Richness (Chao1 index) and evenness (Shannon index) alpha-diversity metrics were not significantly affected by the incubation time or treatment type (Fig. 5C). While the bacterial community remained predominantly Bacteroidota (~ 65%), inulin inclusion increased the relative abundance of Bacillota, Actinomycetota (formerly Actinobacteria), and Euryarchaeota members, particularly at 2 days (Fig. 5D). There was a dose-dependent and temporal-related increase in *Bifidobacterium* and *Lactobacillus* species, as well as other members of the Lactobacillaceae family in response to inulin inclusion (Fig. 5E). Furthermore, inulin supplementation resulted in increased volatile fatty acid concentration ex vivo, where 1.5% inulin favoured the production of propionic and butyric acid, and 3% inulin resulted in an increased molar proportion of acetic acid (Figure S3).

**Fig. 6:**
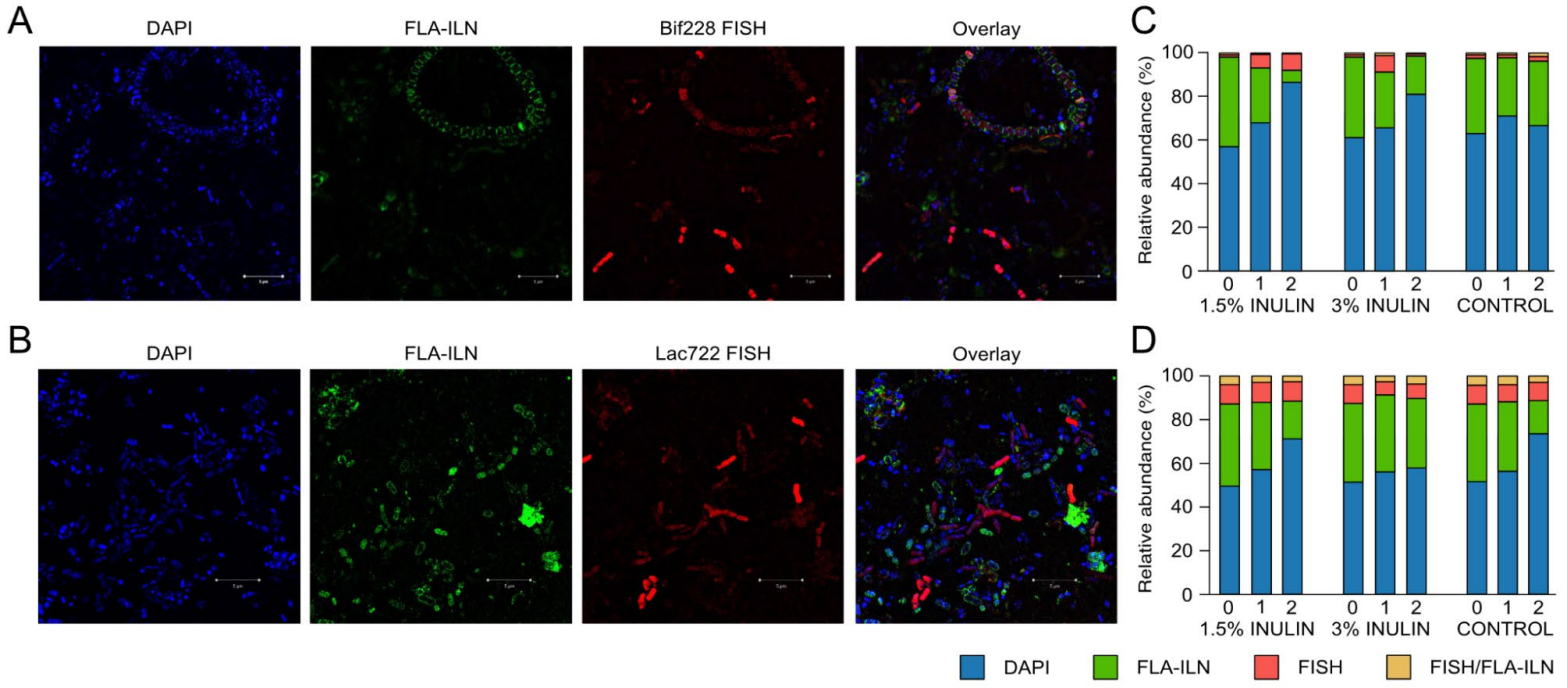
Epifluorescence visualization of FLA-ILN interactions in rumen samples enriched with inulin. **(A)** *Bifidobacterium* members identified using Bif228 FISH probe and **(B)** *Lactobacillus* members identified using Lac722 FISH probe, and their interactions with fluorescently labelled inulin (FLA-ILN) in a 3% inulin-enriched rumen sample taken after 2 d of incubation, and subsequently incubated with 0.2% FLA-ILN for 24 h. Cells were co-stained with DAPI. **(C)** Cell enumeration of rumen microbial communities incubated with FLA-ILN and Bif228, or **(D)** FLA-ILN and Lac722 FISH probe, in cultures sampled over time

The ability of rumen microbiota to metabolize inulin was assessed using FLA-ILN in batch culture samples to determine if pre-exposure to inulin had an influence on FLA-ILN uptake. The total cell density of the starting rumen microbial community was determined by enumerating DAPI-stained cells and was 1.1 × 10^6^ ± 1.1 × 10^5^ mL^− 1^. Within these communities, on average 28.5 ± 9.3% of cells showed uptake of FLA-ILN, whereas 28.9 ± 7.3% of the community was able to utilize inulin when not previously exposed to the prebiotic (Fig. 6C, D).

To investigate correlated taxonomic relationships with inulin supplementation, rumen microbial communities underwent concomitant incubations with FLA-ILN and either Bif228 [33] or Lacto722 [34] FISH probes. This analysis determined that 1.6 ± 0.2% and 8.9 ± 0.2% of the initial rumen bacterial communities were identified as *Bifidobacterium* and *Lactobacillus* species, respectively (Fig. 6A, B). An increase in *Bifidobacterium* abundance occurred within 1 day of inulin supplementation (1.5% inulin: 1.5 ± 0.1% to 6.6 ± 2.9%; 3% inulin: 1.6 ± 0.2% to 7.6 ± 1.9%), where this increase was sustained until the 2 day time point in samples enriched with 1.5% inulin. Some populations of *Bifidobacterium* species showed uptake of FLA-ILN at 1 day; 0.65 ± 0.1% of the 1.5% inulin, and 1.3 ± 0.2% of the 3% inulin-enriched community. In comparison, there was no significant change in the abundance of *Lactobacillus* species as the population stayed between 7 and 9% regardless of the sampling time point or treatment condition. On average, 3.3 ± 0.7% of the *Lactobacillus* cells showed uptake of FLA-ILN. A large proportion of the rumen microbial communities showed FLA-ILN utilization at 0 and 1 day, where there was a decrease in relative abundance at the 2 day time point (Fig. 6C, D). This result seen at 2 days could indicate that the FLA-ILN probe is being digested by cells and removed from the environment, resulting in less staining over time.

### The rumen microbial community associated with the in situ digestion of inulin

An in situ study was performed to evaluate the effect of feeding inulin (2% dry matter basis) on the rumen microbiota. The Shannon diversity index was affected by incubation time (*P* < 0.001; Figure S4); however, both treatment (*P* = 0.6) and the interaction of treatment × time (*P* = 0.2) did not affect Shannon diversity. Similarly, richness was affected by time only (*P* < 0.01; Figure S4). Both alpha-diversity metrics deceased at 12 h of incubation, and then increased at 72 h, compared to the 2 h timepoint. PERMANOVA revealed that ruminal incubation time had the greatest effect on microbial structure (R^2^ = 0.38, *P* = 0.001), followed by treatment (R^2^ = 0.06, *P* = 0.001).

**Fig. 7:**
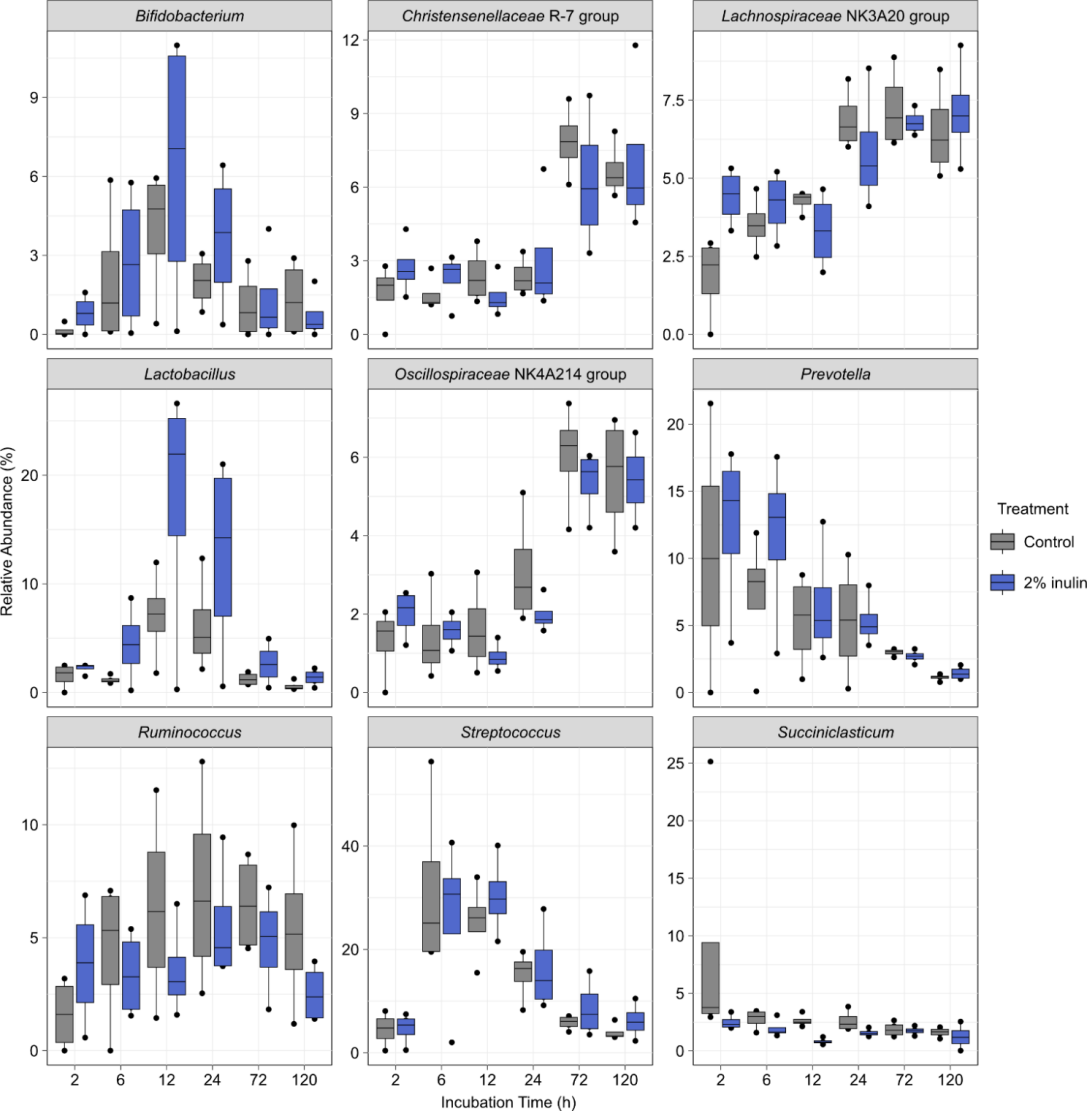
Relative abundance of the dominant genera after in situ digestion. Cows were fed a diet supplemented without (*N* = 4, control) or with inulin (2% dry matter basis; *N* = 4, inulin). Respective diets were incubated in the rumen of cows from 2–120 h and ruminal microbiota were then analyzed. Error bars indicate ± standard error of the mean. The box in the plots indicates the interquartile range (IQR) (middle 50% of the data), the middle line represents the median value, and the whiskers represent 1.5 times the IQR

Across treatments and time, the most abundant phyla were Firmicutes (64.0%), Bacteroidota (16.49%), Actinomycetota (6.51%) and Euryarchaeota (2.12%) (data not shown). In total, 174 genera were observed, with the 9 most abundant being *Streptococcus* (14.6%), *Prevotella* (6.3%), *Lachnospiraceae* NK3A20 group (5.2%), *Lactobacillus* (5.1%), *Ruminococcus* (4.7%), *Christensenellaceae* R-7 group (3.9%), *Oscillospiraceae* NK4A214 group (3.1%), *Bifidobacterium* (2.28%), and *Succiniclasticum* (2.1%) (Fig. 7). For these genera, similar trends were observed between 2% inulin and control treatments, with *Bifidobacterium*, *Lactobacillus* and *Streptococcus* initially increasing, then decreasing in relative abundance; and *Christensenellaceae* R-7 group and *Oscillospiraceae* NK4A214 group increasing across time, and *Prevotella* decreasing in abundance. A total of 67 genera exhibited a significant change (*P* < 0.05, log2(FC) > 2 or log2(FC) < -2) in relative abundance for more than three time points, when inulin-treated cattle were compared to the control group (Figure S5). Except for *Oscillospiraceae* NK4A214 group, each of the top 9 genera differed in abundance between control and 2% inulin groups.

## Discussion

### Structural analysis of cereal crop fructans

Several factors, including genetics, growth stage, and tissue type, influence the abundance of fructans within plants. For mature cereal crops, fructan amounts range from 0.9 to 4.2% (dry matter basis, DMB) in barley [35], and 0.7–2.9% (DMB) in wheat [36]; whereas during kernel development, maximal values of 25.1% and 18.2% (DMB) have been reported for barley and wheat collected 13 DAA, respectively [37]. Here, the fructan abundance varied between cereal crop type and tissue type, where the highest fructan content was within the barley stem section, yet the highest fructan DP average was seen within the winter wheat kernels at 9.1 (Table 1). This average DP is slightly higher than previous reports of 7.3 ± 0.4 (14 DAA) [38], however within this previous study, a maximum DP of 19 was also reported. Further, the presence of 1,2,6-Fru*f* in all six fractions indicated that the purified cereal crop fructans contain graminan-type fructans, which is in agreement with previous reports [7, 11]. Utilization of these in-house purified fructans by the three rumen-derived Bifidobacteria and Lactobacilli was rapid (Fig. 4A) in comparison to the growth of these species on levan and inulin-type fructans (Fig. 2A). This result is consistent with those previously reported, where *Bifidobacterium* and *Lactobacillus* species isolated from the gastrointestinal tract of humans showed rapid growth on agave fructans (DP 3–11), whereas growth on inulin was only achieved by some strains and was delayed [39].

Utilization of small FOS (avg. DP 3–9, Table 1) may be preferred regardless of which glycosidic linkages are present, as the substrates may be directly imported into the cells; whereas for larger DP fructans (i.e., inulin, levan), cleavage of the polysaccharide, and subsequent import, and metabolism, would depend on the enzymatic specificity of extracellular GH32s encoded within their genomes.

### Bacterial catabolism of fructans

Within this study, three rumen-derived *Bifidobacterium* and *Lactobacillus* species were explored for their capability to utilize structurally diverse fructans. Each species was determined to possesses at least one GH32 enzyme associated with fructan metabolism. The genome of *L. vitulinus* was determined to contain the highest number of GH32 enzymes, where one (NZ_JNKN01000017.1_4) was predicted to be secreted and active on levan. FruA, the closest homolog to the NZ_JNKN01000017.1_4 GH32 of *L. vitulinus* also contains a CBM66, and was previously shown to hydrolyze the terminal fructosyl residues of inulin and levan at the non-reducing end [40]. The presence of CBM66 in both GH32s suggests a role in potentiating enzyme function by binding to terminal fructosyl residues [41]. Previously, the removal of CBM66 in the GH32 belonging to *Bacillus subtilis* showed a decrease in GH32 activity against levan, however it still functioned as a nonspecific β-fructosidase [41]. In contrast, as the genomes of *B. boum* and *B. merycicum* were not predicted to encode extracellular inulin-specific GH32s (Fig. 1), utilization of inulin by these two species, which has also been observed in other studies [42, 43], is intriguing. These results may be explained by the inulin substrate being a mixture of DPs [28], with smaller FOS being preferentially degraded first [44, 45]. It has been previously speculated that the primary exponential growth phase is necessary to produce enough β-fructofuranosidase for the breakdown of larger fructan fractions [45].

### Fructan metabolism by ruminal microbial communities

To examine the effects inulin has on ruminal microbial communities, an ex vivo batch culture experiment was conducted where inulin was added at 1.5% or 3% to naïve rumen sample. Within this study, the molar proportions of propionic and butyric acid increased in response to 1.5% inulin, whereas an increase in acetic acid was seen in rumen sample supplemented with 3% inulin (Figure S3). It has previously been reported that acetic acid production requires acetogenic fibers such as inulin and galacto-oligosaccharides as the substrate [46]. There was also a dose-dependent increase in the relative abundance of *Bifidobacterium* and *Lactobacillus* species, amongst other lactic acid bacteria (Fig. 5) seen within the rumen samples supplemented with inulin. Similarly, an increased abundance of Bifidobacteria and Lactobacilli in rumen samples was also seen when dairy cows were fed a diet containing 300 g of inulin per day where this also resulted in increased production of the volatile fatty acids propionate and butyrate [47]. The increased molar proportion of acetic acid seen within the 3% inulin rumen sample within this study, could potentially result from the increased abundance and activity of *Bifidobacterium* and *Lactobacillus* species, as they are both known to produce lactic and acetic acid [48, 49]. Additionally, this higher inclusion rate may support homoacetogenic bacteria present within the ruminal microbial community [50].

Next-generation physiology approaches such as FLAPS can quickly assign cellular phenotypes within complex community samples [20]. Utilization of the FLA-ILN probe showed that over a quarter of the rumen microbial community was able to utilize inulin upon initial exposure (Fig. 6). This suggests that inulin-utilizing bacteria are indigenous in the rumen, perhaps resulting from the presence of fructans consumed when cattle are fed grazing or conventional diets [7, 9, 51]. Further, it was also observed that *Lactobacillus* species within these microbial communities had a higher propensity to utilize inulin and other fructans, in comparison to *Bifidobacterium* species found within the same environment. This could result from the presence of more than one GH32 in the genomes of *Lactobacillus* species, which would provide a more elaborate catalytic toolkit than a single β-fructofuranosidase.

Similar to our ex vivo experiment, ruminal alpha-diversity was not affected by the addition of 2% inulin to cow diets (Figure S4). This is in contrast to a previous study that included inulin in diets fed to dairy cows, and showed that ruminal diversity increased after 3 h of feeding [47]. Though not significant, alpha-diversity was numerically lower in inulin-fed cattle after 12 h of incubation in our study. This coincided with large increases in the relative abundance of both *Lactobacillus* and *Bifidobacterium* members (Fig. 7), suggesting that inulin selected for specific bacteria involved in its degradation and decreased diversity at that timepoint. Thus as inulin is digested, changes in microbiota related to its utilization may alter diversity longitudinally. While *Bifidobacterium* and *Lactobacillus* species were the focus of this study, it is also important to note that other members within rumen microbial communities could potentially utilize inulin and other fructan types as well. For instance, the rumen bacteria *Butyrivibrio fibrisolvens* strain 3071 and *Treponema zioleckii* have been previously shown to utilize levan from Timothy grass [52, 53]. Whereas other rumen bacterial members, such as *Pseudobutyrivibrio ruminis* strain 3 [54], have shown growth on diverse types of fructans (i.e., inulin and levan) [54]. In a similar study, *Muribaculaceae* and *Acetitomaculum* species were also found to increased in relative abundance within the rumen microbiome of cows supplemented with 200 g/d of inulin [55], where these genera were noted for their ability to elevate volatile fatty acids levels. Further, fructan-utilization by some rumen bacterial species may result in the release of large amounts of fructose and FOS into the environment [44, 45], where these by-products could support the growth of bacteria that are unable to utilize the primary fructan substrate [56].

While few studies have evaluated the effect of feeding inulin to ruminants, our in situ results showed similarities to the study conducted by Wang et al. [47]. The authors reported increases in *Bifidobacterium*, *Butyrivibrio*, *Christensenellaceae* R-7, *Lachnospiraceae* NK3A20 group, *Lactobacillus*, and *Prevotella* 3 h after feeding in dairy cattle. Although we also found increases in these genera in the early incubation timepoints (2–6 h), the relative abundances varied at later timepoints compared to the control. Wang and colleagues [47] attributed increases in propionate and butyrate to elevated abundances of *Prevotella* and *Butyrvibrio*. While we did not observe sustained increases in *Prevotella*, *Butyrvibrio* was increased at most timepoints, as were taxa within the *Prevotellaceae* family, which strongly correlates to ruminal propionate concentrations [57]. It was also noteworthy that *Treponema* was reduced at 5/6 timepoints in inulin-fed cattle. This genus includes Gram-negative spirochetes such as *T. denticola*, which can infect abrasions on the feet of cattle causing contagious digital dermatitis [58]. It is interesting to speculate that a reduction in *Treponema* might reduce shedding and subsequently environmental prevalence and the risk of digital dermatitis. However, not all *Treponema* species play a role in the development of digital dermatitis, as *T. bryantii* is a common member of the rumen microbiome that is associated with cellulolytic bacteria [59].When fed at higher levels to dairy cattle, inulin resulted in similar changes in fecal bacteria, compared to those in the rumen, possibly through some inulin bypassing the rumen and being degraded in the lower digestive tract [60]. Thus, it is worthwhile to further evaluate the effect of inulin on total digestive tract microbiota in beef cattle.

Bifidobacteria and Lactobacilli have been associated with improving the health and well-being of monogastric hosts [61, 62]. Yet, it remains unclear whether the prebiotic quality of enhancing these beneficial genera can occur to a significant extent within a ruminant animal model. Results presented here suggest that inulin may have limited prebiotic potential within the rumen microbiome, as supplementation with inulin promoted the proliferation of *Bifidobacterium* and *Lactobacillus* species, along with an increased concentration of volatile fatty acids upon the first 48 h of incubation. However, it is important to note that ex vivo and in situ experimental models have limitations (e.g., accumulation of fermentation end products in batch cultures, focused analysis of the rumen microbial community associated with the feed bags in in situ trials). Future in vivo rumen experiments are warranted to examine these results and if inulin has other effects on beef cattle health and performance.

## Conclusion

The ability of rumen-derived *Bifidobacterium* and *Lactobacillus* species to metabolize fructans was dependent on the fructan DP and linkage composition, and correlated well with the inventory of GH32s encoded in their genomes. All three species could utilize fructans purified from commercial FOS and inulin, and purified from immature cereal crops; however, only *L. vitulinus* was able to proliferate on levan. This observation was supported by the presence of a predicted fructan β-fructosidase active on levan (EC 3.2.1.80). The prebiotic potential of inulin was further assessed in ex vivo naïve rumen microbial communities, where inulin supplementation increased the proliferation of Bifidobacteria and Lactobacilli within 2 days of incubation. This result was also seen in in situ ruminal microbial communities sampled from adult beef cattle at 12 h. Together, results from these studies suggest that co-administering inulin and inulin-utilizing *Lactobacillus* bacteria may represent a beneficial synbiotic for cattle. Further, FLA-ILN/FISH incubations allowed for direct visualization of inulin-utilizing members within the rumen microbial community, including members belonging to *Bifidobacterium* and *Lactobacillus* species. Future work will be required to investigate other inulin-utilizing taxa within the rumen microbial community, as well as investigate whether diverse fructans (i.e., graminan-type) available on farm stimulate beneficial prebiotic, probiotic, and/or synbiotic responses in cattle.

## Methods and materials

### Bioinformatics analysis

Assembled genomes of *B. boum*, *B. merycicum*, and *L. vitulinus* were downloaded from the NCBI assembly database. Whole-genome sequences were run through the dbCAN2 meta server [24] using HMMscan to determine the total CAZyme content for each species. Predicted CAZyme genes for enzymes belonging to the GH32 family were selected and analyzed using the in-house bioinformatics pipeline, SACCHARIS v2 [26], to predict the enzymatic activity of the unknown GH32s belonging to the three species. Sequence and accession numbers of characterized GH32s were extracted from the CAZy database [16] on June 25, 2022, whereby all protein sequences were aligned using MUSCLE v5 [25]. ModelTest-NG [63] was used for best-fit model selection, and the phylogenetic tree was created using FastTree v2 1.11 [64]. Annotation of the GH32 phylogenetic tree was done in R studio [65], using the packages: ggplot2 [66], plyr [67], and treeio [68]. InterProScan 5 [27] and dbCAN2 [24] were used to identify the domain boundaries of each GH32 enzyme.

### Fructan utilization by bovine-derived *Bifidobacterium* and *Lactobacillus* species

#### Bacterial species and culture conditions

Rumen-derived *B. boum* (ATCC 27,917), *B. merycicum* (ATCC 49,391), and *L. vitulinus* (ATCC 27,783) were purchased from CedarLane Laboratories (Burlington, ON, Canada). *Bifidobacterium* species were grown in Trypticase-phytone-yeast extract (TPY) medium [69], and *Lactobacillus* species were grown in de Man, Rogosa and Sharper (MRS) medium, in an anaerobic chamber (atmosphere: 85% N_2_, 10% CO_2_, 5% H_2_) at 37 °C for 24 h. For growth profiles, overnight cultures (OD_600nm_ 1.0-1.3) were centrifuged (4,000 × *g*, 10 min) and washed twice with carb-free (cf.) medium before being diluted to an OD_600nm_ of 0.2 in either 2X cfTPY or 2X cfMRS medium.

1% (w/v) carbohydrate solutions were made for fructose (F0127, Sigma, USA), fructooligosaccharides from chicory (F8052, Sigma, USA), inulin from chicory (I2255, Sigma, USA), levan from *Erwinia herbicola* (L8647, Sigma, USA), and fructans purified in-house from the kernel and stem fractions of winter wheat, spring wheat, and barley (7 DAA; see Sect. 5.3). Wells of a 96-well microtiter plate (Greiner CELLSTAR^®^, Sigma, USA) were filled with 100 µL of sterilized 1% carbohydrate; and 100 µL of bacterial inoculant (OD_600nm_ of 0.2). Negative control wells consisted of 100 µL of 2X cf. medium combined with 100 µL of 1% carbohydrate, and were used to normalize growth curves. Additional media controls were conducted, where the growth of each species was assessed in rich media (TPY or MRS) and the respective cf. media version. Polyurethane Breath-Easy^®^ sealing membranes (Sigma, USA) were used to seal the 96-well plates. Absorbance (OD_600nm_) of each well was measured with a stratus plate reader (Cerillo, USA), and was recorded on a microSD card every 10 min for 48 h. The mean (± standard deviation) for each condition (*N* = 4) was plotted using GraphPad Prism version 9.1.1.

#### FLAPS generation, incubations, and visualization

Fluorescently labelled inulin (FLA-ILN) and levan (FLA-LVN) were generated using a previously described protocol [29] where a mild acid hydrolysis (0.2 M HCl, 20 min at 50 °C) was performed on levan prior to labelling to solubilize the polysaccharide. For the production of fluorescently labelled fructans purified from immature barley kernel and stem fractions (DP < 15), FLA-labelled samples underwent ethanol precipitation (95% ethanol, -20 °C, overnight) followed by centrifugation (4,000 × *g*, 10 min) to remove excess FLA and CNBr. Following freeze-drying, samples were run through a Supelclean™ ENVI-Carb™ SPE column (Sigma, USA) using 80% ethanol to elute purified FLA-BK and FLA-BS.

Rumen-derived *B. boum. B. merycicum*, and *L. vitulinus* were inoculated in either TPY or MRS and grown as described above. Cells were harvested at OD_600nm_ ~ 0.9 and centrifuged (5,000 × *g*, 10 min). Pellets were suspended in 2X cfTPY or 2X cfMRS and washed twice before being resuspended in 2 mL 2X cfTPY or 2X cfMRS media supplemented with 0.5% inulin or 0.5% levan for FLA-ILN or FLA-LVN incubations, respectively. After ~ 16 h of incubation with unlabelled fructan, cultures were centrifuged and washed three times as above, with a final resuspension in 2 mL 2X cfTPY or 2X cfMRS media. 20 µL of the 2X cfTPY or 2X cfMRS resuspension was used as the T0 control, as the cells were not exposed to FLAPS. 40 µL aliquots of each sample were combined with 40 µL of 0.4% FLA-ILN or FLA-LVN, where 20 µL was taken at the 1 h and 1 d time points. For FLA-BK and FLA-BS incubations, the cells were grown in TPY or MRS and were not exposed to 0.5% purified fructans from BK and BS fractions before FLAPS exposure. Cells were immediately fixed with 1 mL 2% formaldehyde overnight at 4 °C. Fixed samples were centrifuged (5,000 × *g*, 10 min), and pellets were washed twice with phosphate saline buffer (PBS, pH 7.4), before being resuspended in 1 mL PBS and stored at 4 °C. Diluted samples were filtered onto a 25 mm, 0.2 μm pore size Isopore™ filter (Sigma, USA) using a gentle vacuum of < 200 mbar. Dried filter pieces were counterstained with 4’6-diamidino-2-phenylindole (DAPI) and mounted on a glass slide using a 4:1 mixture of Citifluor™ AFI mountant solution to Vectashield^®^ vibrance antifade mounting medium. All pure culture samples were visualized using a Leica DM RBE microscope with a cooled 2.8 megapixel camera (Leica DFC 7000T) and a X-Cite 110 LED illumination system with a filter cube containing 365 nm for DAPI and 475 nm for FITC. All samples had an exposure time of 1 s for image capturing.

### Immature cereal crop fructans

#### Collection of immature cereal crops and fructan purification

Above-ground biomass of hard red winter wheat (cv. AAC Coldfront), Canadian prairie spring wheat (cv. AC Andrew), and barley (cv. AC Metcalfe) was collected from experimental plots at the Lethbridge Research and Development Centre (Lethbridge, AB, Canada) 7 DAA in July 2021. Each crop was divided into kernel, stem, and leaf components whereby each section was freeze-dried and ball-milled. Alcohol-insoluble residues were extracted from the ball-milled samples as described by Low et al. [70]. The resulting residue was de-starched using α-amylase (2 U mg^− 1^ sample; Sigma, USA) at 40 °C for 2 h. Fructans were then purified using a fructan assay kit (Megazyme Ltd., Ireland), with slight modifications. α-galactosidase (200 U mL^− 1^, *Aspergillus niger*, Megazyme Ltd., Ireland) was added along with the kit’s sucrase/amylase mixture to remove galactosyl-sucrose oligosaccharides and residual starch. Resulting solutions were run through a Supelclean™ ENVI-Carb™ SPE column (Sigma, USA) according to Jones et al. [71], except 30% ethanol was used to precondition and elute fructans off the column.

#### HPAEC-PAD analysis of fructans purified from immature cereal crop fractions

High-performance anion exchange chromatography (HPAEC) was performed using a Dionex ICS-3000 chromatography system (Thermo Scientific) equipped with an autosampler and a pulsed amperometric detector (PAD). 10 µL of each 1:10 diluted sample (0.2 μm filtered) was injected onto an analytical (3 × 150 mm) PA200 column (Thermo Scientific) and eluted at a 0.5 mL min^− 1^ flow rate with a sodium acetate gradient (0 to 60 min, 10–200 mM) in a constant background of 30 mM NaOH. A 1:10 dilution of 0.1% FOS solution and a standard mixture, composed of 50 µM sucrose, 50 µM 1-kestose, 227 µM fructose, and 227 µM glucose, were run in parallel. Elutions were monitored with the PAD detector (standard quadratic waveform). Data was collected using the Chromeleon chromatography management system and plotted using GraphPad Prism version 9.1.1.

#### LC-MS analysis of fructans purified from immature cereal crop fractions

Purified fructans from winter wheat, spring wheat, and barley kernel and stem fractions were diluted to 250 µM with distilled water and filtered (0.2 μm). Fructan separation from 10 µL injections was performed on a Vanquish ultra-high performance liquid chromatography (UHPLC) system (Thermo Scientific) using a Hypercarb porous graphitic carbon reversed phase HPLC column (3 μm particle size, 2.1 mm x 10 mm; Thermo Scientific). A gradient was run with increasing concentrations of acetonitrile (Table S3) with a flow rate of 400 µL min^− 1^. Fructans separated by UHPLC were detected by electrospray ionization mass spectrometry (ESI-MS) on an Orbitrap Fusion Tribrid system (Thermo Scientific) in negative ion mode. Mass spectra parameters are shown in Table S4. Data were collected in centroid mode and analyzed with Xcalibur 3.1 software Qual Browser (Thermo Scientific).

#### Methylation-GC-MS analysis of fructans purified from immature cereal crop fractions

Freeze-dried fructan sample (~ 5 mg) was dissolved in 1 mL of dimethyl sulfoxide by magnetic stirring overnight at room temperature in a glass tube sealed by a Teflon lined screw cap with head space filled with N_2_. Sodium hydroxide powder (~ 100 mg) and 0.6 mL of methyl iodide were added to the tube, and the mixture was magnetically stirred for 3 h at room temperature with a tube covered with aluminum foil and head space filled with N_2_ [71, 72]. The reaction product was partitioned in 3 mL of dichloromethane and 3 mL of deionized water five times, and each time the upper phase was discarded and the lower phase was left untouched, followed by evaporating to dryness the final lower phase under N_2_. The dried per-O-methylated product was magnetically stirred in 2 mL of 0.5 M trifluoroacetic acid at 50 °C for 2 h with tube head space filled with N_2_, followed by evaporation to dryness by N_2_ [73, 74]. The hydrolysis product was then converted to deuterium labeled partially methylated alditol acetates (PMAAs) by reduction with sodium borodeuteride then per-*O*-acetylation by the heated mixture of acetic anhydride and trifluoroacetic acid (5:1, v/v), as described by the previous report [75], except that anhydrous sodium sulfate instead of anhydrous calcium chloride was used for cleaning up the final products [76]. The PMAAs redissolved in ethyl acetate were analyzed on an Agilent 7890B-5977B GC-MS system (Agilent Technologies, Santa Clara, CA) installed with a medium polarity Supelco SP-2380 capillary column (60 m × 0.25 mm × 0.20 μm, Sigma-Aldrich) with a constant column outlet helium flow rate of 0.8 mL/min. Sample solutions were injected at an inlet temperature of 250 °C with a split ratio of 10:1. The oven temperature was programmed to start at 120 °C (hold 1 min) followed by increasing at 3 °C/min to 200 °C (hold 50 min) then 3 °C/min to 250 °C (hold 20 min). The PMAAs were identified by comparing their MS fragmentation patterns with those of reference derivatives and the literature [77] and quantified based on the GC-MS data according to the published protocol [78]. Two separate experiments were conducted on each sample.

### 5.4 Inulin enrichment in artificial non-adapted rumen systems

#### Rumen sample collection and processing

All animals were cared for in according with the Canadian Council of Animal Care (CCAC, 2009). Rumen sample was collected (Animal Use Protocol number 1004) from a cannulated, non-lactating Angus cow fed a basal diet composed of 50% barley silage and 50% barley grain supplemented with a mineral and vitamin mix. The pure rumen sample was filtered through two layers of cheesecloth prior to being transferred into an anaerobic chamber (atmosphere: 85% N_2_, 10% CO_2_, 5% H_2_; at 37 °C) for ex vivo batch culture experimentation. The sample was equally distributed into three 50 mL falcon tubes, where one contained 1.5% (w/v) Orafti^®^ IPS inulin, the second contained 3.0% (w/v) Orafti^®^ IPS inulin, and the final tube contained no inulin. The tubes were incubated anaerobically for 48 h with occasional mixing. 5-mL aliquots were taken from each tube at 0, 1, and 2 d time points and were used for downstream 16S metagenomics, FLA-ILN/FISH incubations, and volatile fatty acid analysis.

#### 16S rRNA gene sequencing and analysis

From the 5-mL aliquots, 2 mL of each sample was centrifuged (20,000 × *g*, 10 min) and supernatants were removed. DNA from the resulting pellets was extracted using a PowerSoil Pro kit (Qiagen, Germany). Purified DNA samples were sent to Génome Québec (Montréal, QC, Canada) for Illumina MiSeq PE250 16S rRNA sequencing using the primers 515 F – 806R targeting the V4 region. Paired reads were quality trimmed and merged using the BBTools software [79]. After merging, the reads were separated into sample fasta files using the mothur info.fastq command [80]. Clustering and classification of the reads were done through the SILVAngs pipeline using the standard settings and the SSU rRNA seed of the SILVA database release 132 [81]. The output files of the SILVAngs pipeline were used to analyze and plot microbial community profiles.

Community analysis, plotting, and statistics were performed in R studio [65] with the packages: phyloseq [82], picante [83], ggplot2 [66], rioja [84], and vegan [85]. Beta-diversity was determined using Bray-Curtis dissimilarity at the bacterial and archaeal genus level, where permutational multivariate analysis of variance (PERMANOVA) was performed with 999 permutations to estimate a *P*-value for differences amongst the time points and treatment conditions. Alpha-diversity (Shannon and Chao1 indices) was conducted, with a pairwise Wilcoxon signed-rank test used for statistical difference evaluation. For the ex vivo experiment, triplicate technical replicates of each sample were used for the 16S sequencing analysis, there were no significant differences between replicates and only the average analysis was used for plotting purposes. For the in situ experiment, pooled samples from each animal were used for analysis. For the in situ study, the 9 genera with the highest relative abundances were plotted, and differentially abundant genera were identified with DESeq2. This was performed by fitting a negative binomial model to the equation “inulin vs. the control” (~ Treatment) at each individual timepoint. Genera of amplified sequence variants that differed in abundance (*P* < 0.01) for more than three time points were visualized in a heatmap.

#### FLA-ILN/FISH incubations and visualization

1 mL from the 1.5% inulin, 3.0% inulin, and control rumen ex vivo batch cultures were added to 1 mL of 0.4% FLA-ILN and incubated anaerobically at 37 °C. From each FLAPS incubation, aliquots were collected at 0 h (prior to FLA-ILN addition), 1 h, and 1 d time points. Samples were fixed and filtered as described above. Dried filter pieces were used for FISH staining.

For FISH, the Bif228 (5’-GATAGGACGCGACCCCAT-3’) [33] and Lacto722 (5’-YCACCGCTACACATGRAGTTCCACT-3’) [34] oligo-nucleotide probes targeting *Bifidobacterium* and *Lactobacillus* respectively, were covalently labelled with four ATTO590 fluorochromes (Integrated DNA Technologies, USA), prior to use. FISH was performed with slight alterations to the protocol of Manz *et* al. [86]. The hybridization buffer contained 900 mM NaCl, 20 mM Tris-HCl (pH 7.5), 0.01% sodium dodecyl sulfate, and a formamide concentration of either 20% or 5% for the Bif228 and Lacto722, respectively. Hybridizations were performed within a 46 °C chamber for 3 h before undergoing a subsequent 15 min wash at 48 °C in a buffer containing 20 mM Tris-HCl (pH 7.5), 2 nM EDTA (pH 8.0), 0.01% sodium dodecyl sulfate, and a NaCl concentration of either 215 mM or 630 mM for the Bif228 and Lacto722, respectively. After FISH, filter pieces were counterstained with DAPI and mounted onto a glass slide as described above. Samples were visualized and enumerated using super-resolution structured illumination imaging as previously described [23].

#### Measurement of volatile fatty acid production

For VFA analysis, 1.5 mL of the 5 mL aliquots was directly added to 0.3 ml 25% meta-phosphoric acid (Fisher Scientific A280) on ice. Samples were mixed and stored at -20 °C until gas chromatographic analysis was performed [87]. VFA data was plotted in GraphPad Prism version 9.1.1 and statistically analyzed using multiple t tests.

### The rumen microbial community associated with in situ digestion of inulin

This study was approved by the Animal Care Committee of Lethbridge Research and Development Centre (Animal Use Protocol number 2107). Ruminally cannulated beef cows (Angus × Herford cross) were used to examine the effects of dietary supplementation with inulin on the rumen microbiota. Control cows (*N* = 4) were fed a mixed ration, once daily, containing alfalfa hay (50%), barley silage (35%), barely grain (12%) and mineral/vitamin supplement (3%). Inulin-treatment cows (*N* = 4) were fed the same diet, but with 2% inulin supplemented (dry matter basis, Orafti^®^ IPS inulin). The diets were formulated to meet the nutrient requirements according to the NRC (2000) and were fed once daily as a total mixed ration. The animals were adapted to the diets by stepping up the amount fed gradually over a 7-day period. Days 1–15 of the experimental period were the adaptation phase, while days 16–20 were the sampling phase.

Approximately 5 g of respective diet sample was ground and then weighed into triplicate Dacron bags and incubated in each cow for 2 h, 6 h, 12 h, 24 h, 72 h, and 120 h. At each time point, triplicate bags were retrieved and processed to analyze rumen microbiota. After retrieval from the rumen, bags were gently rinsed under running tap water to remove external food particles, and then freeze-dried to evaluate microbiota associated with feed particles. For each animal, digestive content in triplicate bags were pooled into a single sample at each time point, ball-milled, and DNA was extracted for analysis of the 16 S rRNA gene, as described above in 4.4.2.

## Electronic supplementary material

Below is the link to the electronic supplementary material.

Supplementary Material 1

Supplementary Material 2

Supplementary Material 3

Supplementary Material 4

Supplementary Material 5

Supplementary Material 6

## Data Availability

The 16S rRNA datasets generated and analyzed during this study are deposited in the NCBI repository under the BioProject No PRJNA1096758 (https://dataview.ncbi.nlm.nih.gov/object/PRJNA1096758?reviewer=pjvtunppnhrftfbmrj78su5109) and PRJNA1101962 (https://dataview.ncbi.nlm.nih.gov/object/PRJNA1101962?reviewer=5ikg7ne3pjh44lh2p5qtlvocu5). Fraser A, 2022. SACCHARIS_2.0 Github https://github.com/saccharis/SACCHARIS_2 (Retrieved June 25, 2022) Hitch TCA, Wylensek D, Clavel T. 2020. Data from *Bifidobacterium boum* strain WCA-130-P53-4B, whole genome shotgun sequencing project. GenBank https://www.ncbi.nlm.nih.gov/nuccore/JABAGJ000000000.1 (accession no. JABAGJ000000000.1) Varghese N, Submissions S. 2016. Data from *Bifidobacterium merycicum* DSM 6492, whole genome shotgun sequencing project. GenBank https://www.ncbi.nlm.nih.gov/nuccore/FQTX00000000.1 (accession no. FQTX00000000.1) Kelly W, Huntemann M, Han J, Chen A, Kyrpides N, Mavromatis K, Markowitz V, Palaniappan K, Ivanova N, Schaumberg A, Pati A, Liolios K, Nordberg HP, Canto MN, Hua SX, Woyke T. 2014. Data from *Kandleria vitulinia* DSM 20405, whole genome shotgun sequencing project. GenBank https://www.ncbi.nlm.nih.gov/nuccore/JNKN00000000.1 (accession no. JNKN00000000.1)
