# Supplementary figures and images for: In vitro and ex vivo metabolism of chemically diverse fructans by bovine rumen Bifidobacterium and Lactobacillus species
Source: Anim Microbiome. 2024 Sep 9;6:50. doi: 10.1186/s42523-024-00328-1 (PMC11382395; doi:10.1186/s42523-024-00328-1)

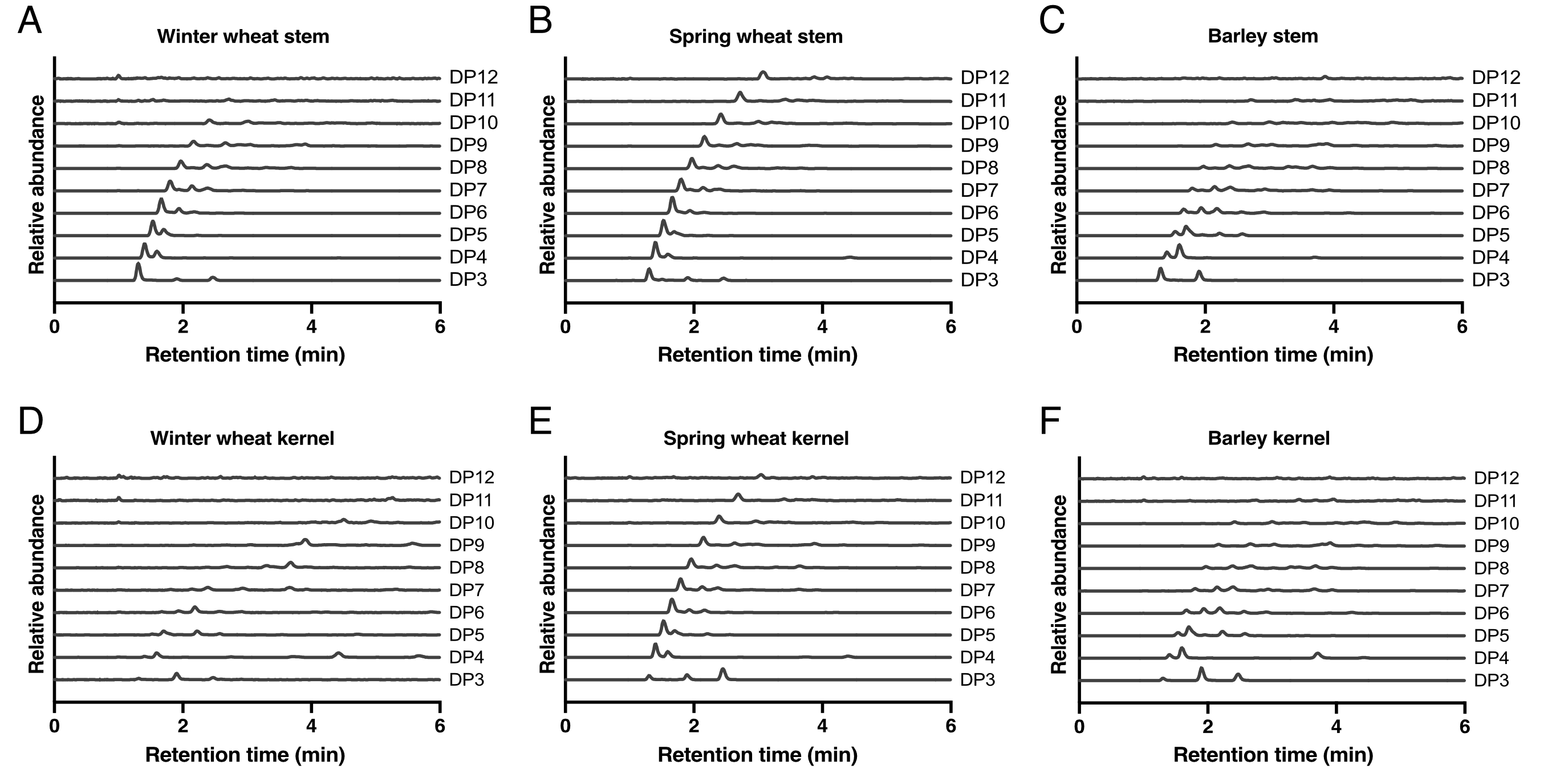

Supplement: Supplementary file 1 — Supplementary Material 1 [file 42523_2024_328_MOESM1_ESM.tiff]

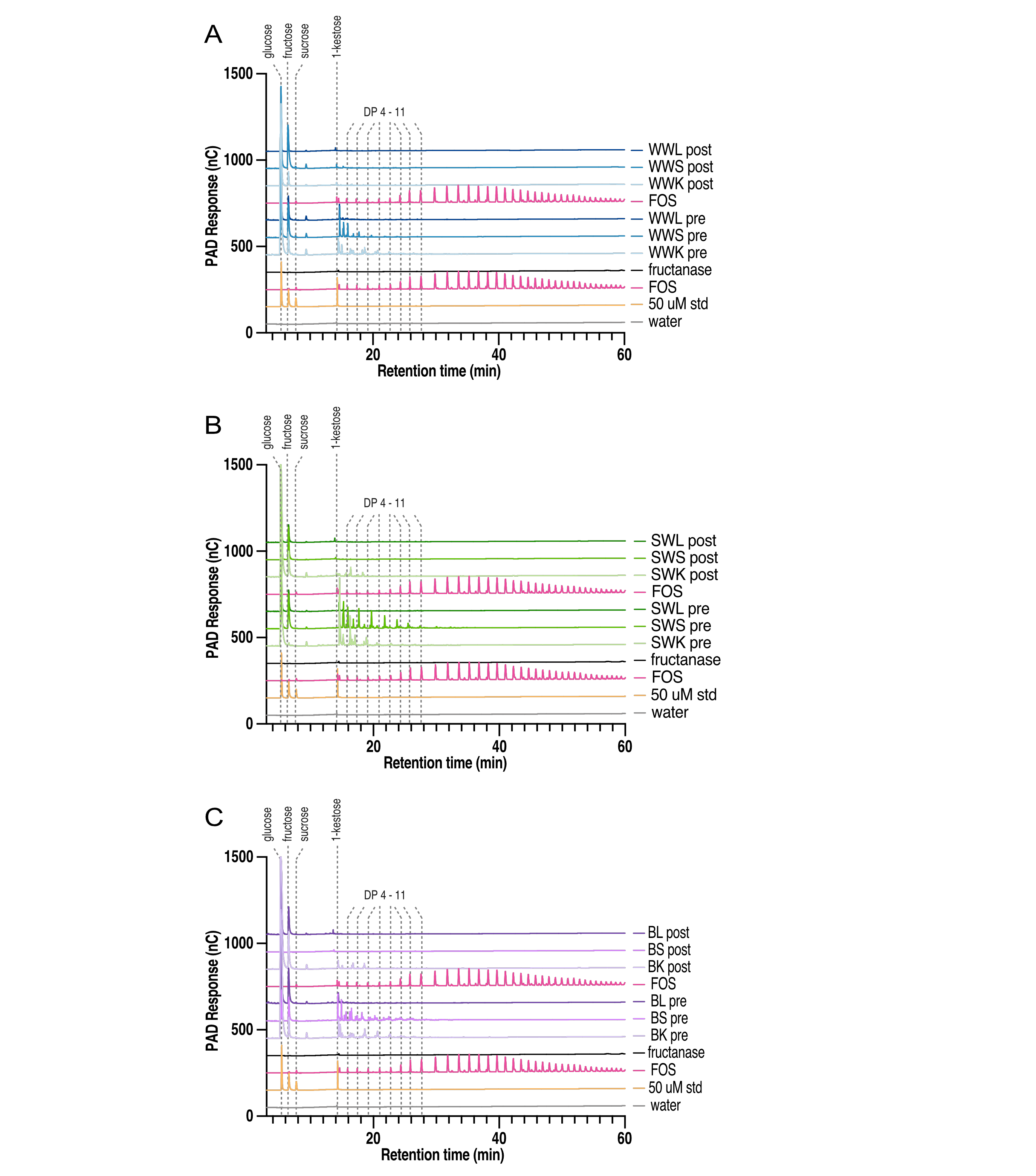

Supplement: Supplementary file 2 — Supplementary Material 2 [file 42523_2024_328_MOESM2_ESM.tiff]

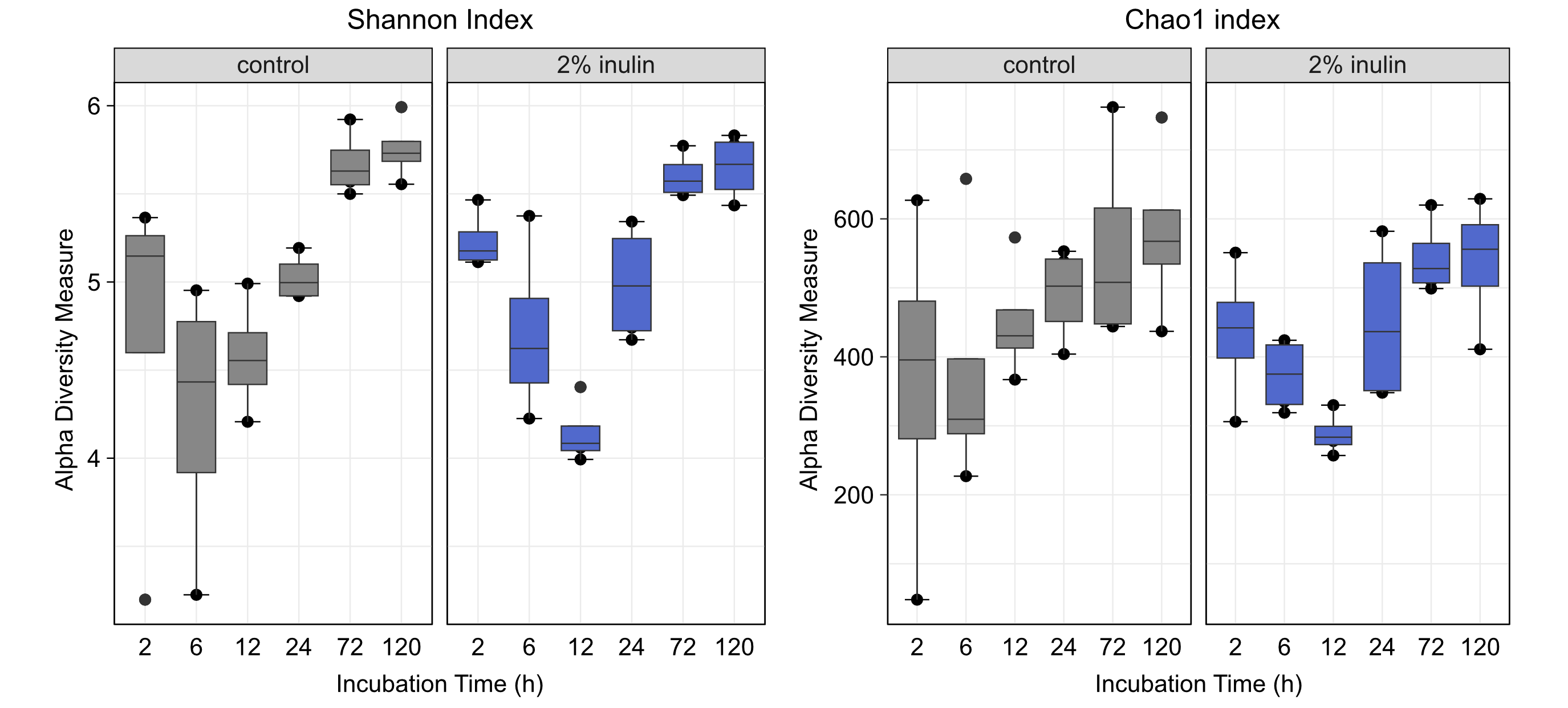

Supplement: Supplementary file 3 — Supplementary Material 3 [file 42523_2024_328_MOESM3_ESM.tiff]

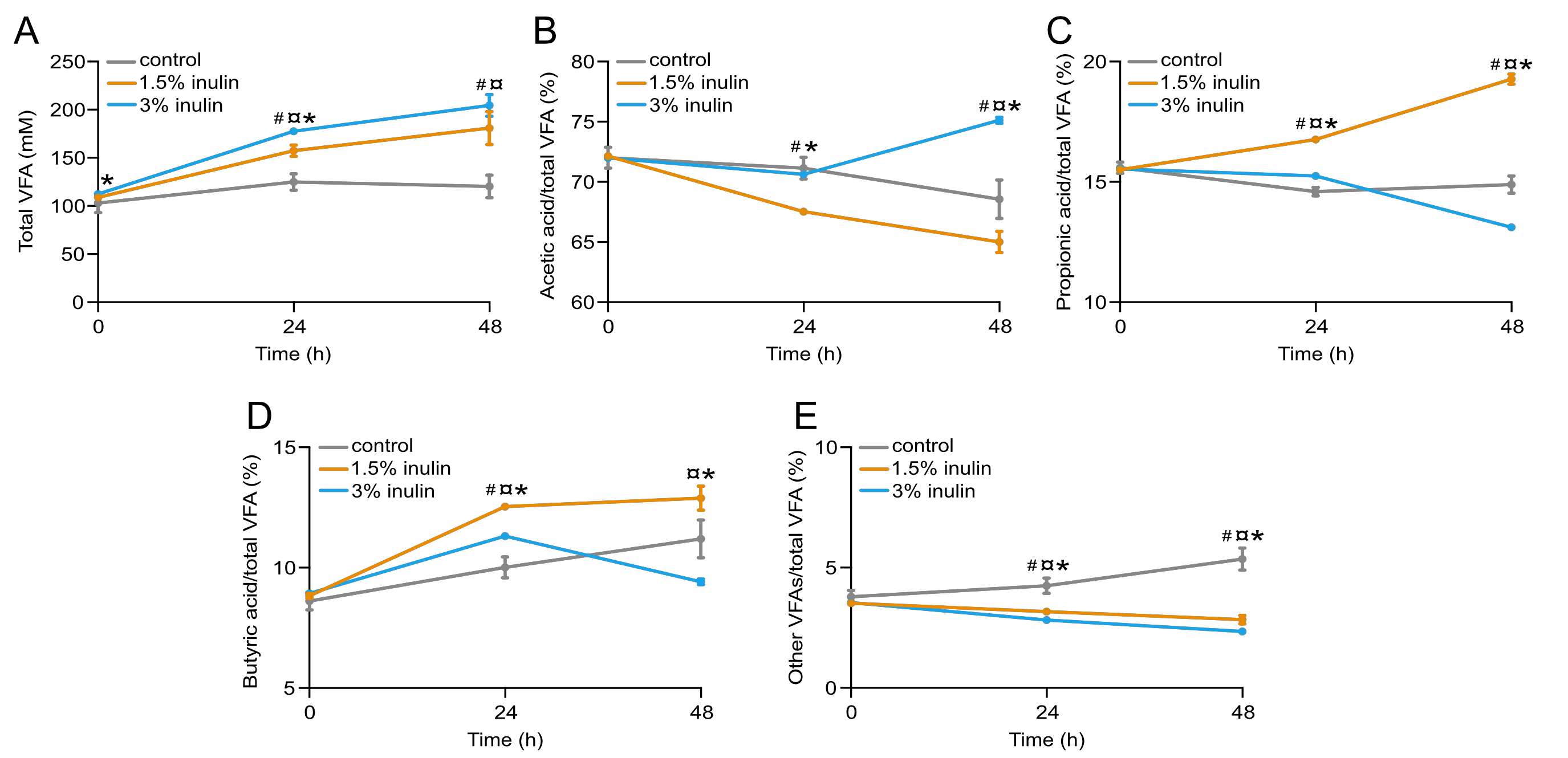

Supplement: Supplementary file 4 — Supplementary Material 4 [file 42523_2024_328_MOESM4_ESM.tiff]

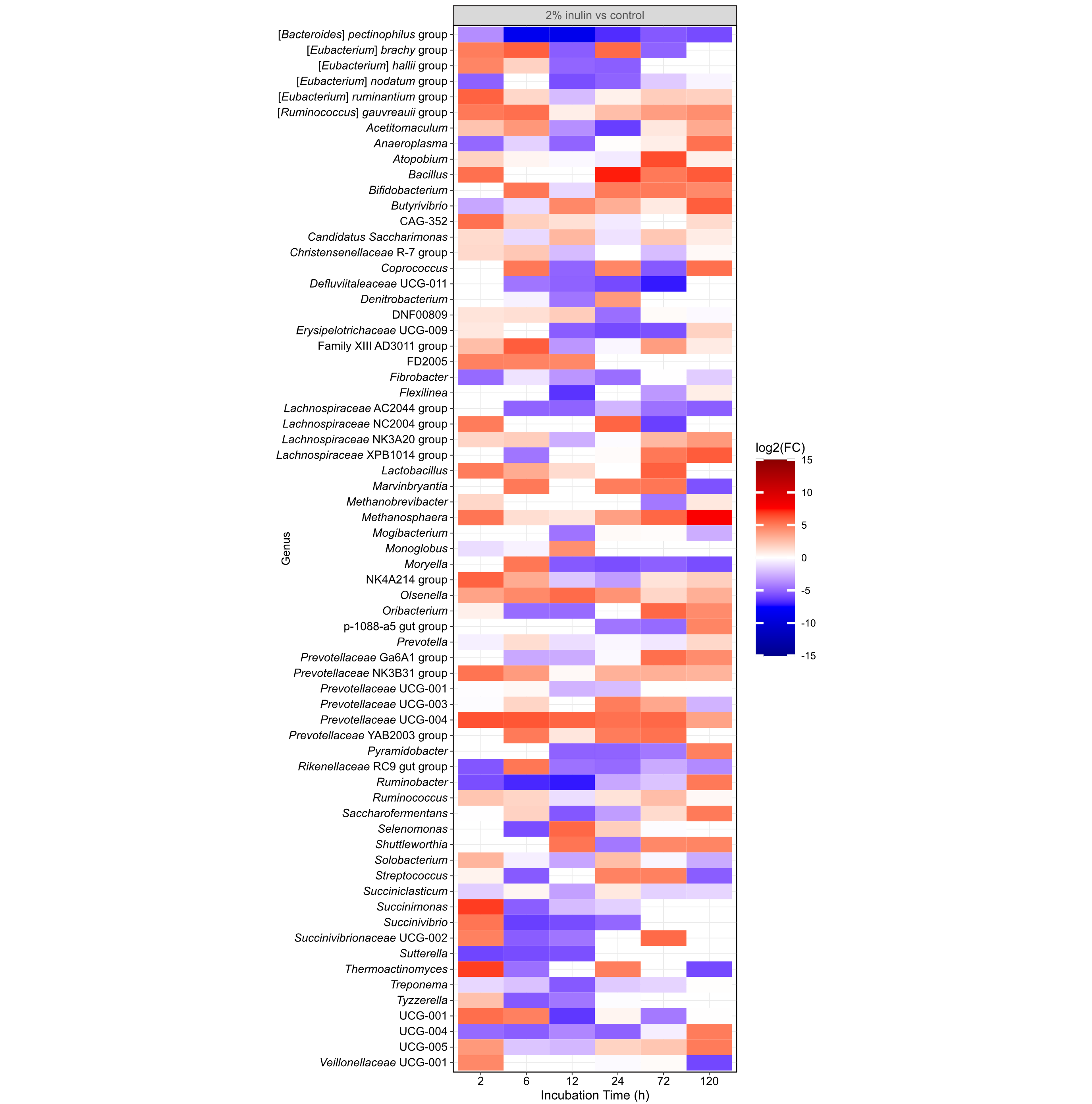

Supplement: Supplementary file 5 — Supplementary Material 5 [file 42523_2024_328_MOESM5_ESM.tiff]
