# Supplementary material for: In vitro and ex vivo metabolism of chemically diverse fructans by bovine rumen Bifidobacterium and Lactobacillus species
Source: Anim Microbiome. 2024 Sep 9;6:50. doi: 10.1186/s42523-024-00328-1 (PMC11382395; doi:10.1186/s42523-024-00328-1)
Supplement: Supplementary file 6 — Supplementary Material 6 [file 42523_2024_328_MOESM6_ESM.docx]

**Supplementary Table 1 Percent identity matrix of the GH32 amino acid sequences from rumen-derived *Bifidobacterium* and *Lactobacillus* species.** Matrix was generated using MUSCLE (25), where grey shading represents sequence conservation > 50%.

|  | **1** | **2** | **3** | **4** | **5** | **6** | **7** | **8** |
| --- | --- | --- | --- | --- | --- | --- | --- | --- |
| **1** | - - - | 51.3 | 20.3 | 26.2 | 20.0 | 22.9 | 20.4 | 20.4 |
| **2** | 51.3 | - - - | 21.3 | 27.1 | 21.0 | 21.2 | 19.8 | 19.8 |
| **3** | 20.3 | 21.3 | - - - | 26.5 | 21.5 | 20.0 | 22.2 | 22.4 |
| **4** | 26.2 | 27.1 | 26.5 | - - - | 25.5 | 25.6 | 25.4 | 26.1 |
| **5** | 20.0 | 21.0 | 21.5 | 25.5 | - - - | 28.6 | 27.3 | 28.0 |
| **6** | 22.9 | 21.2 | 20.0 | 25.6 | 28.6 | - - - | 52.4 | 50.5 |
| **7** | 20.4 | 19.8 | 22.2 | 25.4 | 27.3 | 52.4 | - - - | 63.5 |
| **8** | 20.4 | 19.8 | 22.4 | 26.1 | 28.0 | 50.5 | 63.5 | - - - |

1 = *B. boum* (NZ_JABAGJ010000007.1_104)

2 = *B. merycicum* (NZ_FQTX01000003.1_36)

3 = *L. vitulinus* (NZ_JNKN01000017.1_4)

4 = *B. merycicum* (NZ_FQTX01000001.1_82)

5 = *L. vitulinus* (NZ_JNKN01000049.1_5)

6 = *L. vitulinus* (NZ_JNKN01000007.1_60)

7 = *L. vitulinus* (NZ_JNKN01000025.1_18)

8 = *L. vitulinus* (NZ_JNKN01000025.1_23)

**Supplementary Table 2 Identification and linkage assignment of deuterium labelled partially methylated alditol acetates (PMAAs) prepared from fructans extracted from the stems and kernels of immature winter wheat, spring wheat, and barley (7 DAA).**

| **Peak**  **No.** | **Relative**  **retention time** | **Deuterium labeled partially methylated alditol acetates** | **Characteristic fragment ions (m/z)** | **Deduced**  **linkages** |
| --- | --- | --- | --- | --- |
| 1 | 0.61 | 2,5-di-*O*-acetyl-(2-deuterio)-1,3,4,6-tetra-*O*-methyl mannitol | 87, 101, 102, 129, 145, 146, 161, 162, 205, 206 | t-Fru*f* |
| 2 | 0.61 | 2,5-di-*O*-acetyl-(2-deuterio)-1,3,4,6-tetra-*O*-methyl glucitol | 87, 101, 102, 129, 145, 146, 161, 162, 205, 206 | t-Fru*f* |
| 3 | 0.68 | 1,5-di-*O*-acetyl-(1-deuterio)-2,3,4,6-tetra-*O*-methyl glucitol | 87, 101, 102, 118, 129, 145, 161,162, 205 | t-Glc*p* |
| 4 | 0.89 | 1,2,5-tri-*O*-acetyl-(2-deuterio)-3,4,6-tri-*O*-methyl mannitol | 87, 101, 129, 161, 190, 205, 234 | 1,2-Fru*f* |
|  |  | 2,5,6-tri-*O*-acetyl-(2-deuterio)-1,3,4-tri-*O*-methyl mannitol | 87, 101, 129, 162, 189, 206, 233 | 2,6-Fru*f* |
| 5 | 0.90 | 2,5,6-tri-*O*-acetyl-(2-deuterio)-1,3,4-tri-*O*-methyl glucitol | 87, 101, 129, 162, 189, 206, 233 | 2,6-Fru*f* |
| 6 | 0.91 | 1,2,5-tri-*O*-acetyl-(2-deuterio)-3,4,6-tri-*O*-methyl glucitol | 87, 101, 129, 161, 190, 205, 234 | 1,2-Fru*f* |
| 7 | 1.00 | 1,5,6-tri-*O*-acetyl-(1-deuterio)-2,3,4-tri-*O*-methyl glucitol | 87, 102, 118, 129, 162, 173, 189, 206, 233 | 1,6-Glc*p* |
| 8 | 1.52 | 1,2,5,6-tetra-*O*-acetyl-(2-deuterio)-3,4-di-*O*-methyl mannitol | 87, 129, 189, 190, 233, 234 | 1,2,6-Fru*f* |
| 9 | 1.53 | 1,2,5,6-tetra-*O*-acetyl-(2-deuterio)-3,4-di-*O*-methyl glucitol | 87, 129, 189, 190, 233, 234 | 1,2,6-Fru*f* |

Note: Retention times are relative to 1,5,6-tri-*O*-acetyl-(1-deuterio)-2,3,4-tri-*O*-methyl glucitol. Due to the symmetry nature of the mannitol structure, two PMAAs cannot be separated by GC and coelute as Peak #4.

**Supplemental Table 3 UHPLC gradient for separation of fructans.**

| **Time**  **(min)** | **A (%)**  **(water)** | **B (%)**  **(acetonitrile)** | **C (%)**  **(2-propanol)** |
| --- | --- | --- | --- |
| 0 | 99 | 1 |  |
| 6 | 90 | 10 |  |
| 16 | 88 | 12 |  |
| 30 | 84 | 16 |  |
| 55 | 62 | 28 | 10 |
| 55.1 | 0 | 80 | 20 |
| 60 | 0 | 80 | 20 |
| 61 | 99 | 1 |  |
| 66 | 99 | 1 |  |

**Supplemental Table 4** **Parameters for ESI-MS on the Orbitrap Fusion Tribrid.**

| **Parameter (units)** | **Value** |
| --- | --- |
| *ESI* |  |
| Spray voltage: negative ion (V) | 2500 |
| Sheath Gas (Arb) | 45 |
| Aux Gas (Arb) | 10 |
| Sweep Gas (Arb) | 1 |
| Ion Transfer Tube Temp (°C) | 325 |
| Vaporizer Temp (°C) | 250 |
|  |  |
| *MS* |  |
| Detector Type | Orbitrap |
| Orbitrap Resolution | 120K |
| Mass Range | Normal |
| Scan Range (m/z) | 150-2000 |
| RF Lens (%) | 50 |
